# Supplementary material for: Proximity labelling of pro-interleukin-1α reveals evolutionary conserved nuclear interactions
Source: Nat Commun. 2024 Aug 8;15:6750. doi: 10.1038/s41467-024-50901-0 (PMC11310415; doi:10.1038/s41467-024-50901-0)
Supplement: Supplementary file 1 — Supplementary Information [file 41467_2024_50901_MOESM1_ESM.pdf]

Supplementary Information

Proximity labelling of pro-interleukin-1α reveals evolutionary conserved nuclear interactions

Rose Wellens[1,2,3], Victor S. Tapia[1,2,3], Paula I. Seoane[1,2,3], Hayley Bennett[4], Antony Adamson[4], Graham Coutts[1,2,3], Jack Rivers-Auty[5], Martin Lowe[6], Jack P. Green[1,2,3], Gloria Lopez-Castejon[3,7], David Brough*[1,2,3] & Christopher Hoyle*[1,2,3]

[1] Division of Neuroscience, School of Biological Sciences, Faculty of Biology, Medicine and Health, University of Manchester, Manchester Academic Health Science Centre, Manchester, M13 9PT, UK.

[2] Geoffrey Jefferson Brain Research Centre, The Manchester Academic Health Science Centre, Northern Care Alliance NHS Group, University of Manchester, Manchester, M13 9PT, UK.

[3] The Lydia Becker Institute of Immunology and Inflammation, University of Manchester, Manchester, M13 9PT, UK.

[4] Genome Editing Unit, Faculty of Biology, Medicine and Health, University of Manchester, Manchester, M13 9PT, UK

[5] Tasmanian School of Medicine, College of Health and Medicine, University of Tasmania, Hobart, Tasmania, 7000, Australia

[6] Division of Molecular and Cellular Function, School of Biological Sciences, Faculty of Biology, Medicine and Health, University of Manchester, Manchester Academic Health Science Centre, Manchester, M13 9PT, UK.

[7] Division of Infection, Immunity and Respiratory Medicine, School of Biological Sciences, Faculty of Biology, Medicine and Health, University of Manchester, Manchester Academic Health Science Centre, Manchester, M13 9PT, UK.

*Corresponding authors. Emails: christopher.hoyle@manchester.ac.uk, david.brough@manchester.ac.uk

Supplementary Table 1. Pro-IL-1α-TurboID significantly enriched proteins. 56 proteins significantly enriched via pro-IL-1α-TurboID-mediated biotinylation. Significance was calculated by two-sample two-tailed t-test of log_2-transformed LFQ intensity values (significance determined as s0=2; FDR=0.01) using Perseus computational platform (n=4). Fold change and p-value were calculated following comparison with TID control. Table shows gene name, protein name, log_2(fold change) and log_{10}(p-value). Source data are provided as a Source Data file.

Protein symbol	Protein name	log_2(fold change)	$-log_{10}$(p-value)
IL1A	Interleukin-1 alpha	11.16	5.67
EP300	Histone acetyltransferase p300	2.72	4.54
YEATS2	YEATS domain-containing protein 2	2.60	4.41
CRTC2	CREB-regulated transcription coactivator 2	2.92	4.30
DMAP1	DNA methyltransferase 1-associated protein 1	2.03	4.30
ZFHX3	Zinc finger homeobox protein 3	2.12	4.18
ARID2	AT-rich interactive domain-containing protein 2	1.72	3.69
KMT2D	Histone-lysine N-methyltransferase 2D	2.26	3.61
ZZZ3	ZZ-type zinc finger-containing protein 3	4.32	3.56
SMTN	Smoothelin	1.96	3.48
QSER1	Glutamine and serine-rich protein 1	1.89	3.43
TRPS1	Zinc finger transcription factor Trps1	2.71	3.31
MYBL2	Myb-related protein B	2.39	3.24
JMJD1C	Probable JmjC domain-containing histone demethylation protein 2C	2.32	3.22
NCOA3	Nuclear receptor coactivator 3	2.71	3.18
MAML1	Mastermind-like protein 1	2.51	3.04
CIC	Protein capicua homolog	1.99	3.00
SUPT20H	Transcription factor SPT20 homolog	2.17	2.95
ARNT	Aryl hydrocarbon receptor nuclear translocator	2.43	2.94
KMT2C	Histone-lysine N-methyltransferase 2C	5.03	2.88
BCORL1	BCL-6 corepressor-like protein 1	1.77	2.79
SMARCE1	SWI/SNF-related matrix-associated actin-dependent regulator of chromatin subfamily E member 1	2.80	2.68
ATN1	Atrophin-1	1.60	2.63
KDM3A	Lysine-specific demethylase 3A	1.81	2.59
BCL9L	B-cell CLL/lymphoma 9-like protein	1.86	2.58
HIVEP1	Zinc finger protein 40	2.27	2.53

ARID1A	AT-rich interactive domain-containing protein 1A	1.73	2.52
NCOA5	Nuclear receptor coactivator 5	2.18	2.48
EP400	E1A-binding protein p400	1.63	2.40
C15orf39	Uncharacterized protein C15orf39	2.07	2.38
ASXL2	Putative Polycomb group protein ASXL2	1.97	2.32
EPC1	Enhancer of polycomb homolog 1	2.13	2.28
BCL9	B-cell CLL/lymphoma 9 protein	2.32	2.27
ELMSAN1	ELM2 and SANT domain-containing protein 1	1.97	2.12
UBR2	E3 ubiquitin-protein ligase UBR2	1.66	2.11
MAD1L1	Mitotic spindle assembly checkpoint protein MAD1	1.91	2.08
POLDIP3	Polymerase delta-interacting protein 3	2.28	2.08
IRF2BP1	Interferon regulatory factor 2-binding protein 1	2.03	2.05
CFAP20	Cilia- and flagella-associated protein 20	1.98	2.03
SAP130	Histone deacetylase complex subunit SAP130	2.88	1.97
SETD1B	Histone-lysine N-methyltransferase SETD1B	4.55	1.96
JUND	Transcription factor jun-D	3.37	1.87
SRSF2	Serine/arginine-rich splicing factor 2	1.84	1.82
KIAA0907	UPF0469 protein KIAA0907	1.80	1.81
GSE1	Genetic suppressor element 1	2.36	1.75
NCOA2	Nuclear receptor coactivator 2	2.27	1.67
FOXK2	Forkhead box protein K2	2.08	1.67
ARL6IP1	ADP-ribosylation factor-like protein 6-interacting protein 1	1.98	1.62
IRF2BP2	Interferon regulatory factor 2-binding protein 2	2.73	1.61
RAB10	Ras-related protein Rab-10	3.53	1.58
PSMB4	Proteasome subunit beta type-4	1.89	1.58
PCYT1A	Choline-phosphate cytidylyltransferase A	1.90	1.54
RNF40	E3 ubiquitin-protein ligase BRE1B	2.11	1.53
GTF2A1	Transcription initiation factor IIA subunit 1	2.64	1.49
NCBP2	Nuclear cap-binding protein subunit 2	2.25	1.42
MGA	MAX gene-associated protein	2.06	1.41

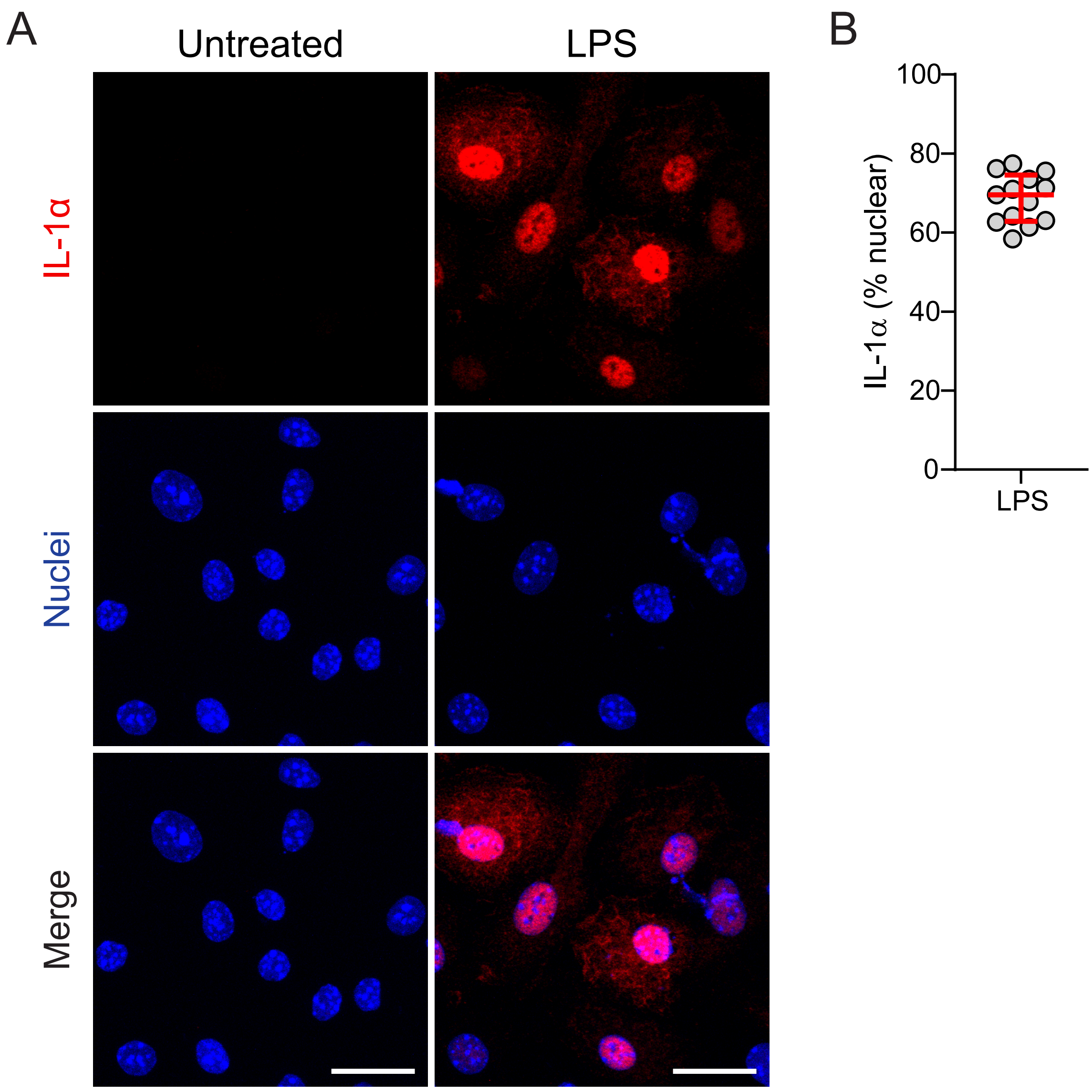

Supplementary Figure 1. Pro-IL-1α localises to the nucleus in primary mouse bone marrow-derived macrophages. Primary mouse bone marrow-derived macrophages were untreated or primed with LPS (1 µg ml^{-1}) for 4 hours. (**A**) Representative maximum projection confocal immunofluorescence images are shown. (**B**) Quantification of IL-1α nuclear localisation in response to LPS priming from full fields of view (n=13 fields of view from three independent experiments). Data are median ± IQR. Scale bars are 25 µm. Source data are provided as a Source Data file.

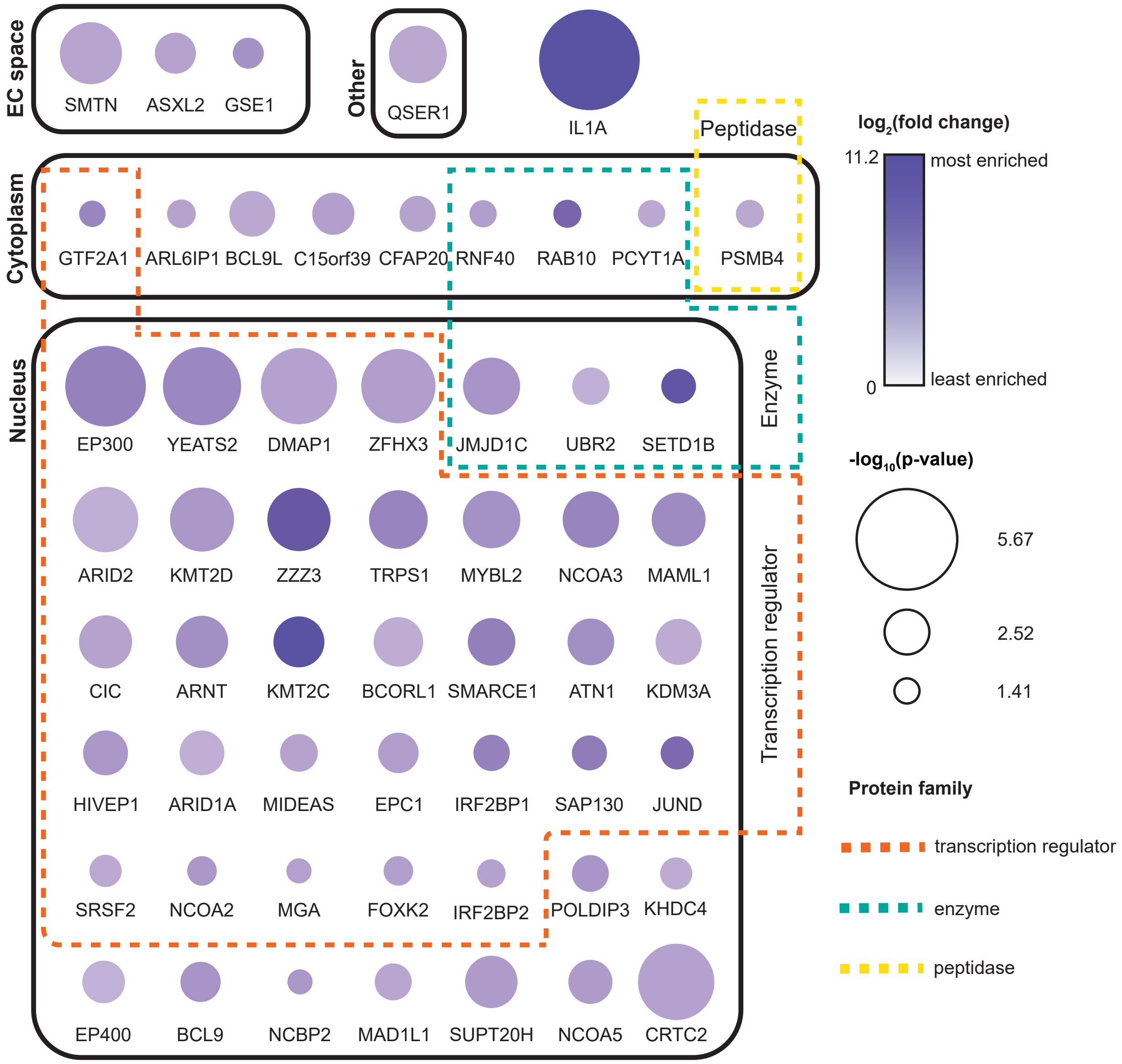

Supplementary Figure 2. Subcellular location and protein family groups of pro-IL-1α-TurboID biotinylated proteins. Proteins were clustered by subcellular location and protein family as described by IPA. Those proteins with no assigned protein family group were categorised as "other" by IPA. Proteins are coloured by fold change in enrichment, displayed as $\log_2$(fold change), and sized by p-value, displayed as $-\log_{10}$(p-value). Significance was determined by two-sample two-tailed t-test of $\log_2$-transformed LFQ intensity values (significance determined as s0=2; FDR=0.01) using Perseus computational platform (n=4). Fold change and p-value were calculated following comparison with TID control. Subcellular locations were determined by IPA and grouped by solid boxes (EC space: extracellular space). Protein families are grouped by dashed lines; transcription regulator (orange); enzyme (green); peptidase (yellow). See Supplementary Table 1.

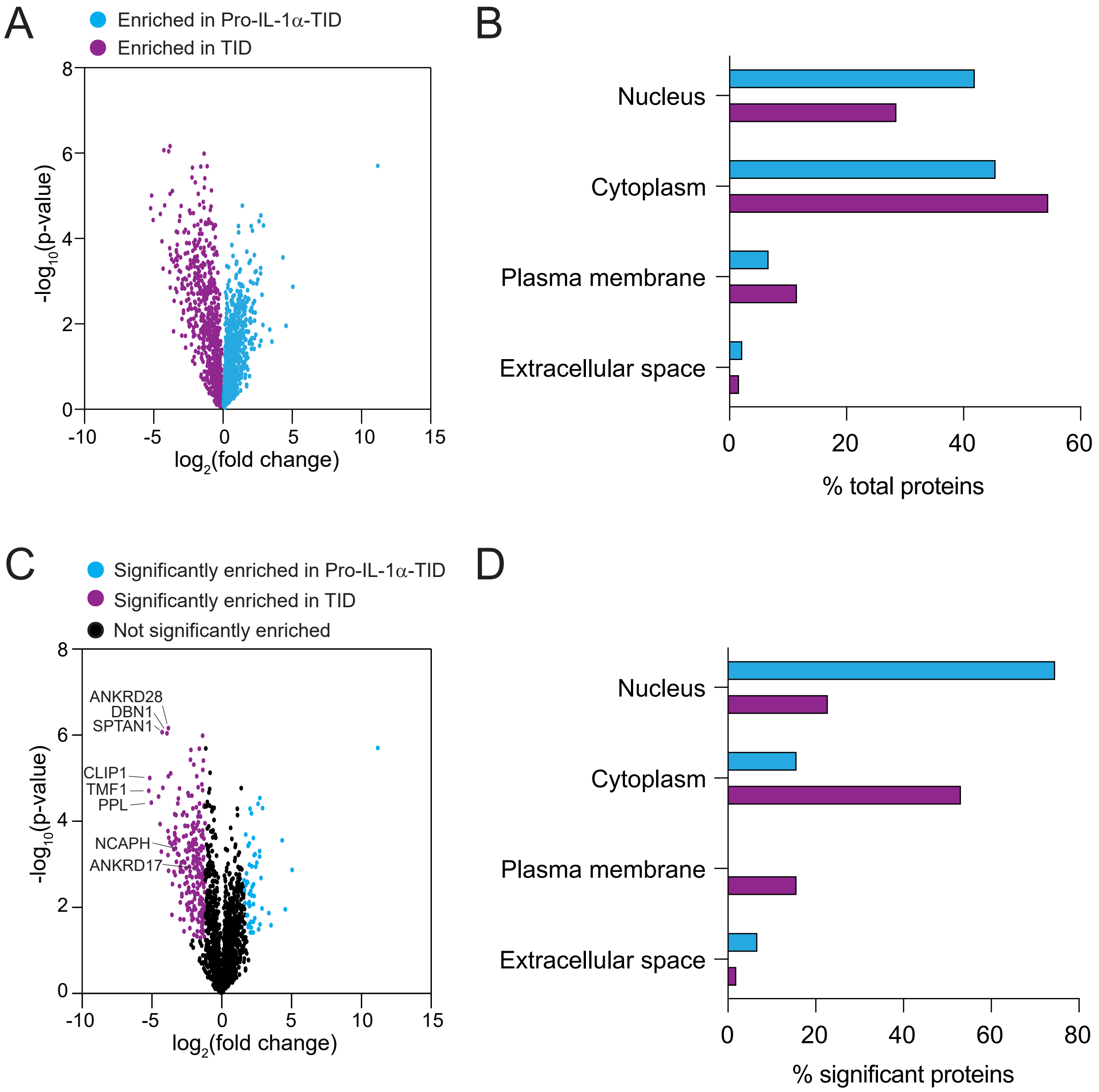

Supplementary Figure 3. Subcellular location of pro-IL-1α-TurboID and TurboID enriched proteins. (**A**) Volcano plot highlighting Pro-IL-1α-TID and TID enriched proteins. (**B**) Subcellular location of Pro-IL-1α-TID and TID enriched proteins. (**C**) Volcano plot highlighting Pro-IL-1α-TID and TID significantly enriched proteins, following two-sample two-tailed t-test of log_2-transformed LFQ intensity values (significance determined as s0=2; FDR=0.01) using Perseus computational platform (n=4). (**D**) Subcellular location of Pro-IL-1α-TID and TID significantly enriched proteins. Source data are provided as a Source Data file.

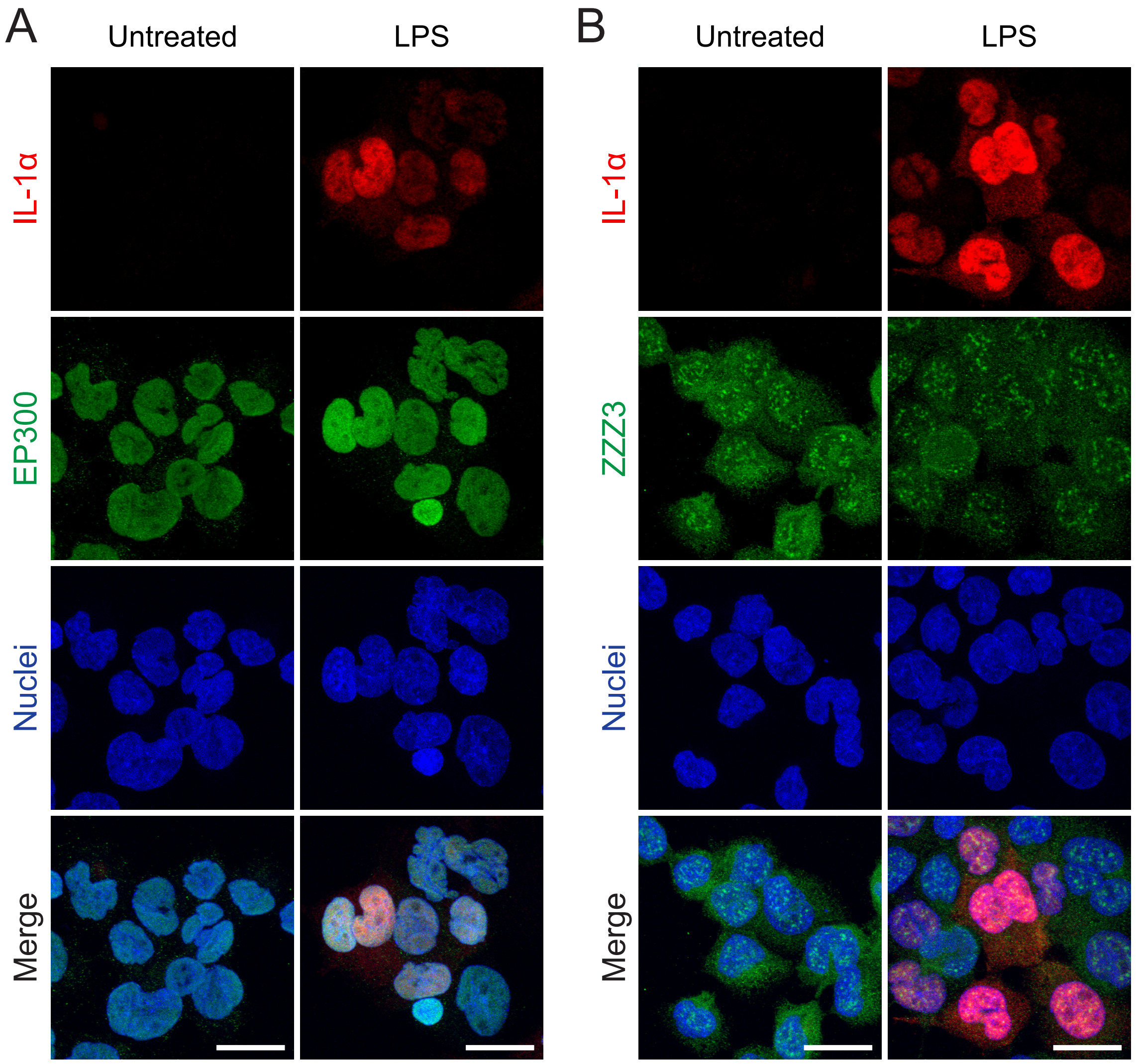

Supplementary Figure 4. EP300 and ZZZ3 co-localise with pro-IL-1α in the nucleus of THP-1 macrophages. THP-1 macrophages were untreated or primed with LPS (1 μg ml^{-1}) for 4 hours, and analysed by immunofluorescence microscopy for pro-IL-1α and (**A**) EP300 or (**B**) ZZZ3 (n=3). Representative maximum projection confocal immunofluorescence images are shown. Scale bars are 20 μm.

A

Species	Amino acid sequence														NLS score
**Intact NLS**															
Human	G	K	V	L	K	K	R	R	L	S	L	-	S	Q	9.5
Chipmanzee	G	K	V	L	K	K	R	R	L	S	L	-	S	Q	9.5
House mouse	G	K	I	L	K	K	R	R	L	S	F	-	S	E	10.5
Norway rat	G	K	I	L	K	K	R	R	L	S	F	-	N	Q	10.5
Pig	G	K	I	L	K	K	R	R	L	S	L	-	N	Q	10
Cow	G	K	I	L	K	K	R	R	L	S	L	-	N	Q	10
Dog	G	K	I	L	K	K	R	R	L	S	L	-	S	Q	10
Rabbit	G	K	I	L	K	K	R	R	L	S	L	-	N	Q	9.5
Common vampire bat	G	K	V	L	K	K	R	R	L	S	F	-	N	Q	10.5
Western European hedgehog	G	K	I	L	K	K	R	R	F	S	L	-	N	Q	9
African savanna elephant	G	K	I	L	K	K	R	R	L	S	L	-	N	Q	9.5
Southern two-toed sloth	G	E	V	L	K	K	R	R	L	S	L	-	S	Q	7
Malayan pangolin	G	K	V	L	K	K	R	R	L	S	L	-	N	Q	10
Minke whale	G	K	I	L	K	K	R	R	L	S	L	-	N	Q	10
Nine-banded armadillo	G	K	V	L	K	R	R	R	L	S	L	-	N	L	11.5
Natal long-fingered bat	G	K	I	L	K	R	R	R	L	T	F	-	N	Q	10.5
Grey short-tailed opossum	R	K	V	K	K	K	R	R	-	P	F	K	N	H	8
**NLS mutants**															
Common bottlenose dolphin	G	K	I	L	K	N	R	W	L	S	L	-	N	Q	<2
Killer whale	G	K	I	L	K	N	R	W	L	S	L	-	N	Q	<2
Naked mole-rat	G	T	V	L	K	K	R	W	L	S	L	-	N	Q	<2
Ord's kangaroo rat	G	K	I	L	K	K	R	L	L	S	L	-	N	Q	<2
Banner-tailed kangaroo rat	G	K	I	L	K	K	R	L	L	S	L	-	N	Q	<2
Southern grasshopper mouse	G	K	I	L	K	K	R	L	L	G	F	-	G	T	<2
Big brown bat	G	K	I	L	K	K	R	W	L	S	F	-	N	Q	<2
American beaver	G	K	I	L	K	K	R	W	L	S	L	-	N	Q	<2
Chinese pangolin	G	K	V	L	K	K	R	W	L	S	L	-	N	Q	<2
Tasmanian devil	K	K	K	E	K	K	K	Q	I	S	E	-	S	H	3
Koala	Q	E	I	E	K	K	R	Q	L	T	R	-	R	H	<2
Common brushtail	R	K	T	-	E	N	R	R	L	F	V	-	S	H	<2
**Consensus sequence from full alignment**															
	G	K	I	L	K	K	R	R	L	S	L	-	N	Q	

KKRR motif amino acid:

- match to human
- synonymous
- non-synonymous to K/R
- non-synonymous

B

Species	Nucleotide sequence												Motif
	K			K			R			R			KKRR
**Intact NLS**													
Human	a	a	g	a	a	g	a	g	a	c	g	g	KKRR
Chipmanzee	a	a	g	a	a	g	a	g	a	c	g	g	KKRR
House mouse	a	a	g	a	a	g	a	g	a	c	g	g	KKRR
Norway rat	a	a	g	a	a	g	a	g	a	c	g	g	KKRR
Pig	a	a	g	a	a	g	a	g	a	c	g	g	KKRR
Cow	a	a	g	a	a	g	a	g	a	c	g	g	KKRR
Dog	a	a	g	a	a	g	a	g	a	c	g	g	KKRR
Rabbit	a	a	g	a	a	a	a	g	a	c	g	c	KKRR
Common vampire bat	a	a	g	a	a	g	a	g	a	c	g	g	KKRR
Western European hedgehog	a	a	g	a	a	g	a	g	a	a	g	g	KKRR
African savanna elephant	a	a	g	a	a	a	a	g	a	c	g	a	KKRR
Southern two-toed sloth	a	a	g	a	a	g	c	g	g	c	g	a	KKRR
Malayan pangolin	a	a	g	a	a	g	a	g	a	c	g	g	KKRR
Minke whale	a	a	g	a	a	g	a	g	a	c	g	g	KKRR
Nine-banded armadillo	a	a	g	a	g	g	a	g	a	c	g	a	KRRR
Natal long-fingered bat	a	a	g	a	g	g	a	g	a	c	g	g	KRRR
Grey short-tailed opossum	a	a	g	a	a	g	a	g	g	c	g	t	KKRR
**NLS mutants**													
Common bottlenose dolphin	a	a	g	a	a	c	a	g	a	t	g	g	KNRW
Killer whale	a	a	g	a	a	c	a	g	a	t	g	g	KNRW
Naked mole-rat	a	a	g	a	a	g	a	g	a	t	g	g	KKRW
Ord's kangaroo rat	a	a	g	a	a	g	a	g	a	t	t	g	KKRL
Banner-tailed kangaroo rat	a	a	g	a	a	g	a	g	a	t	t	g	KKRL
Southern grasshopper mouse	a	a	g	a	a	g	a	g	a	c	t	g	KKRL
Big brown bat	a	a	g	a	a	g	a	g	a	t	g	g	KKRW
American beaver	a	a	g	a	a	g	a	g	a	t	g	g	KKRW
Chinese pangolin	a	a	g	a	a	g	a	g	a	t	g	g	KKRW
Tasmanian devil	a	a	g	a	a	g	a	a	a	c	a	a	KKKQ
Koala	a	a	g	a	a	g	a	g	a	c	a	a	KKRQ
Common brushtail	g	a	a	a	a	t	a	g	a	c	g	a	ENRR

KKRR motif nucleotide:

- match to human
- synonymous
- non-synonymous to K/R
- non-synonymous

Supplementary Figure 5. Several mammalian species have acquired mutations in the pro-IL-1α NLS KKRR motif, resulting in predicted loss of nuclear localisation. (**A**) Pro-IL-1α nuclear localisation sequence (NLS) amino acid sequences of several mammalian species, including those that have lost predicted NLS function. Amino acid sequences and the consensus sequence were taken from the alignment of IL-1α from all isoforms of all 157 species (see Supplementary Data 1). NLS scores were estimated using NLSmapper. Sequences correspond to G78-Q90 in human sequence. (**B**) KKRR motif nucleotide sequences for each of the species. Synonymous (green), non-synonymous (red), and non-synonymous to K/R (orange) mutations are highlighted.

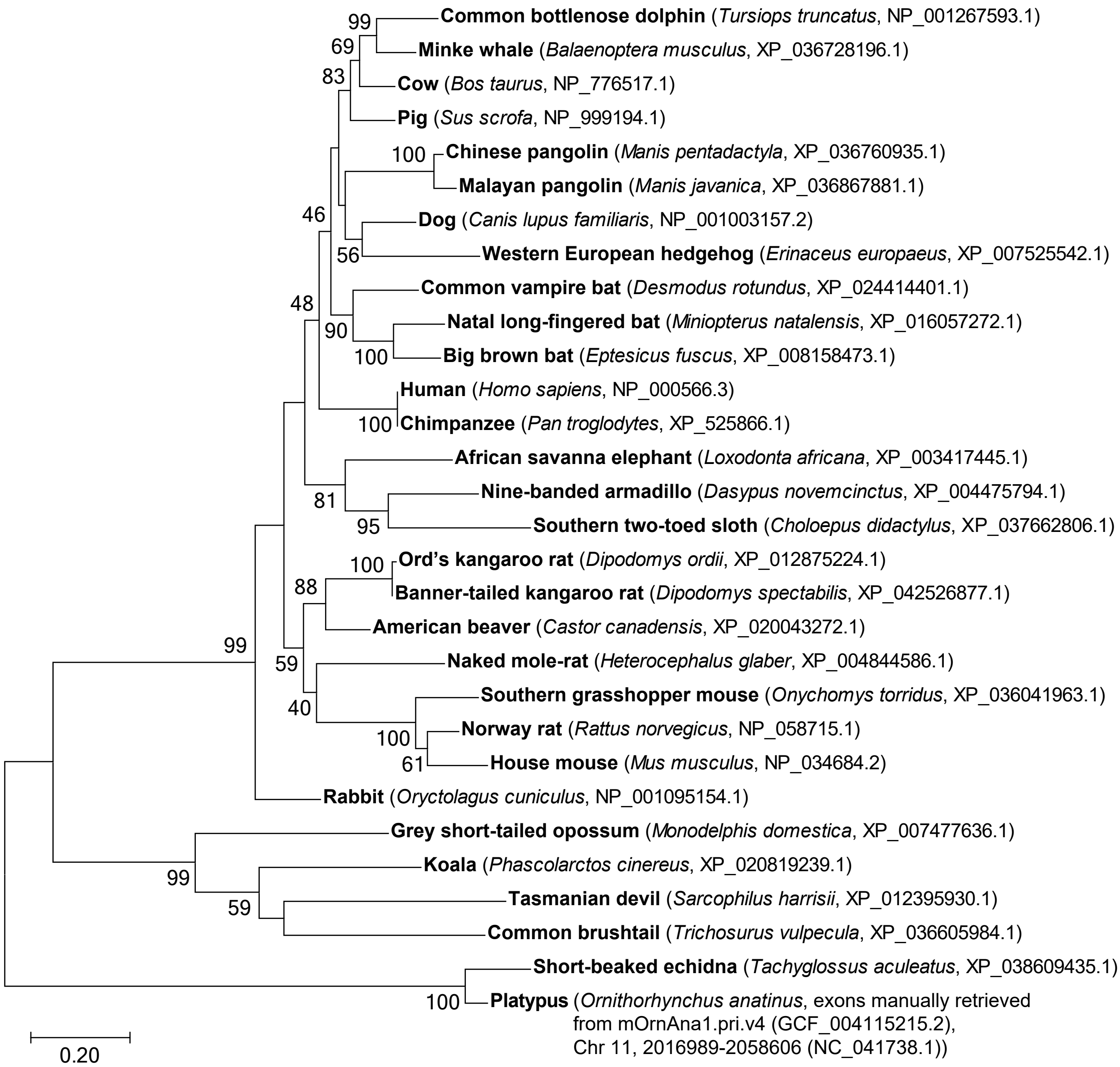

Supplementary Figure 6. Phylogenetic tree analysis of IL-1α. IL-1α amino acid sequences from species shown in Figure 4A and the platypus and short-beaked echidna were used to generate a phylogenetic tree using MEGA X. The evolutionary history was inferred by using the Maximum Likelihood method and JTT matrix-based model[1]. The tree with the highest log likelihood (-7883.54) is shown. The percentage of trees in which the associated taxa clustered together is shown next to the branches, from 1000 bootstrap replications[2]. Values of less than 40 are not shown. The tree is drawn to scale, with branch lengths measured in the number of substitutions per site. This analysis involved 30 amino acid sequences. All positions with less than 95% site coverage were eliminated, i.e., fewer than 5% alignment gaps, missing data, and ambiguous bases were allowed at any position (partial deletion option). There were a total of 248 positions in the final dataset.

A

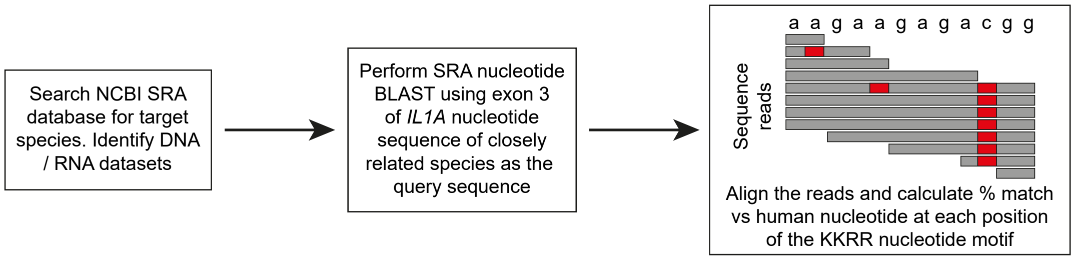

B

Species	Nucleotide sequence												Motif	NLS score
	K			K			R			R			KKRR	
American beaver	a	a	g	a	a	g	a	g	a	t	g	g	KKRW	<2
Eurasian beaver	a	a	g	a	a	g	a	g	a	t	g	g	KKRW	<2
Desmarest's spiny pocket mouse	a	a	a	a	a	g	a	g	a	t	g	g	KKRW	<2
Pacific pocket mouse	a	a	g	a	a	g	a	g	a	t	g	g	KKRW	<2
Bailey's pocket mouse	a	a	g	a	a	g	a	g	a	t	g	g	KKRW	<2
Rock pocket mouse	a	a	g	a	a	g	a	g	a	t	g	g	KKRW	<2
Stephen's kangaroo rat	a	a	g	a	a	g	a	g	a	t	t	g	KKRL	<2
Merriam's kangaroo rat	a	a	g	a	a	g	a	g	a	t	t	g	KKRL	<2
Ord's kangaroo rat	a	a	g	a	a	g	a	g	a	t	t	g	KKRL	<2
Banner-tailed kangaroo rat	a	a	g	a	a	g	a	g	a	t	t	g	KKRL	<2
Botta's pocket gopher	a	a	g	a	a	a	a	g	a	t	g	g	KKRW	<2

match to human — synonymous — non-synonymous

C

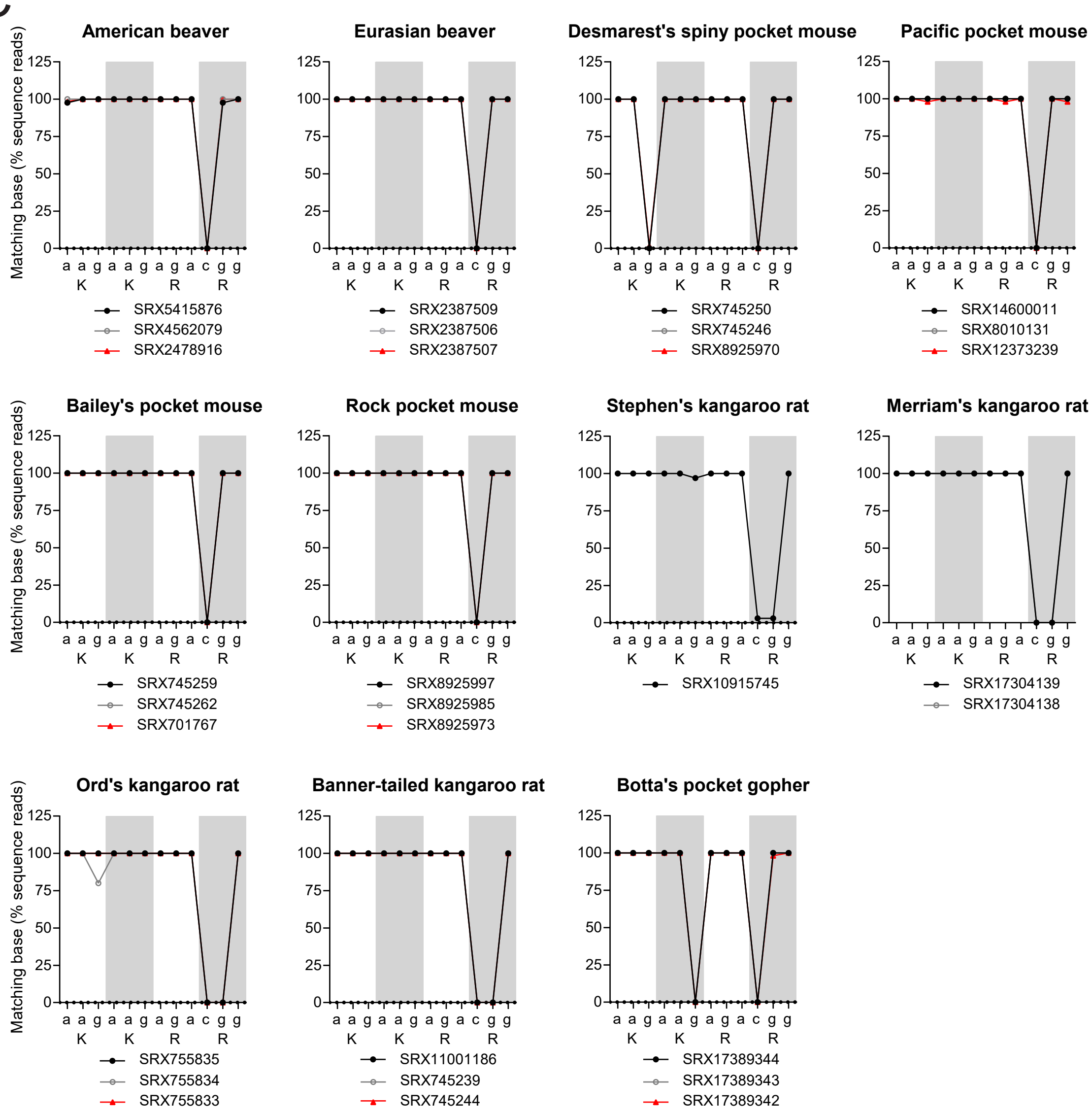

Supplementary Figure 7. Assessment of nuclear localisation sequence (NLS) mutations in the rodent suborder castorimorpha. (**A**) Pipeline for retrieving and analysing sequence reads from sequence read archive (SRA) database to assess KKRR motif nucleotide similarity to human sequence. (**B**) Nucleotide sequences of the KKRR motif of the castorimorpha species, only including species for which an *IL1A* sequence could be retrieved from the sequence read analysis. (**C**) Sequence reads from the SRA database, which were retrieved by performing sequence read nucleotide BLAST with the exon three nucleotide sequence of the American beaver or Ord's kangaroo rat used as the query sequence. Data are presented as % sequence reads that match the human KKRR nucleotide sequence 'aagaagagacgg'. Where possible, reads were retrieved from at least three individuals, from three separate studies. See Supplementary Data 2. Synonymous (green), non-synonymous (red), and non-synonymous to K/R (orange) mutations are highlighted.

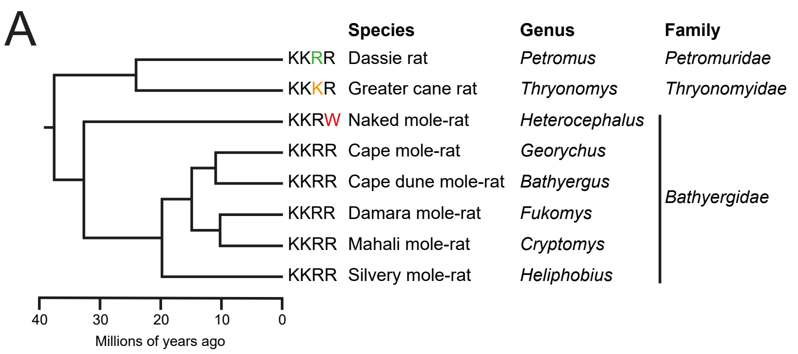

B

Species	Nucleotide sequence												Motif	NLS score
	K			K			R			R			KKRR	
Dassie rat	a	a	g	a	a	g	a	g	g	c	g	g	KKRR	9.5
Greater cane rat	a	a	g	a	a	g	a	a	g	c	g	g	KKKR	3.5
Naked mole-rat	a	a	g	a	a	g	a	g	a	t	g	g	KKRW	<2
Cape mole-rat	a	a	g	a	a	g	a	g	a	c	g	g	KKRR	10
Cape dune mole-rat	a	a	g	a	a	g	a	g	a	c	g	g	KKRR	10
Damaraland mole-rat	a	a	g	a	a	g	a	g	a	c	g	g	KKRR	10
Mahali mole-rat	a	a	g	a	a	g	a	g	a	c	g	g	KKRR	10
Silvery mole-rat	a	a	g	a	a	g	a	g	a	c	g	g	KKRR	9.5

match to human synonymous non-synonymous to K/R non-synonymous

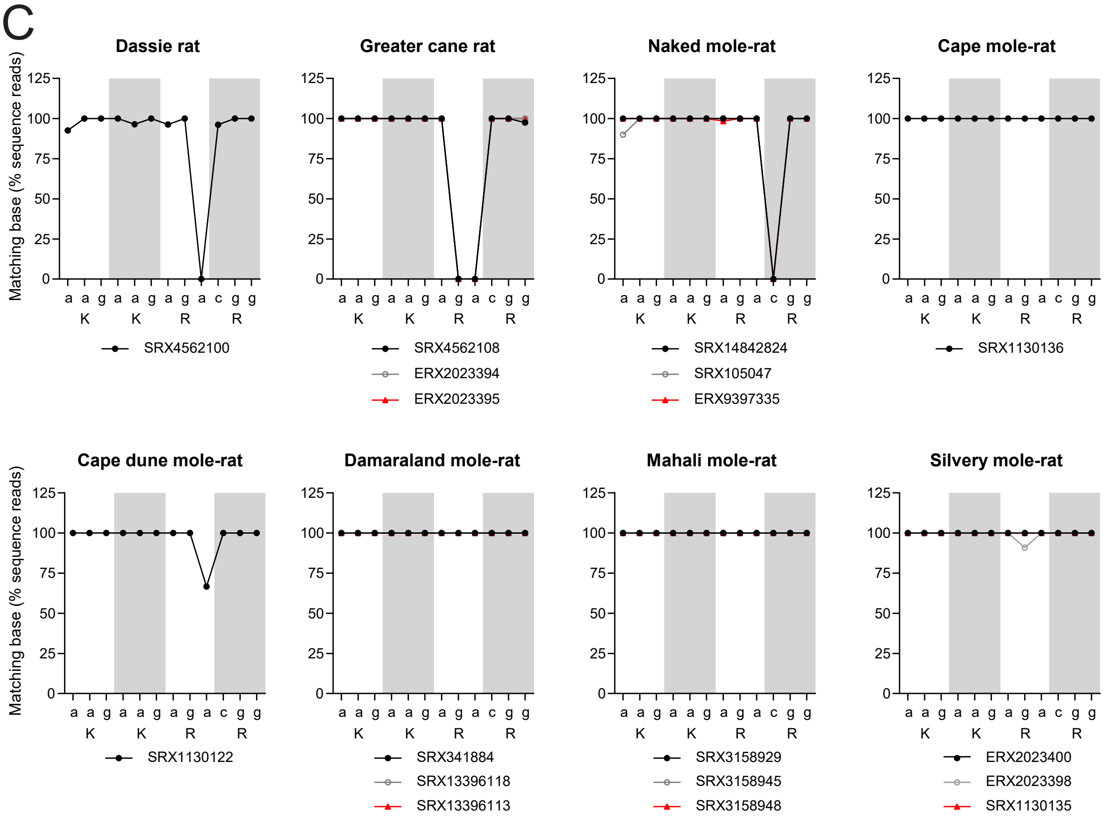

Supplementary Figure 8. Assessment of nuclear localisation sequence (NLS) mutations in the naked mole-rat superfamily. (**A**) Model evolutionary tree of the naked mole-rat superfamily, only including species for which an *IL1A* sequence could be retrieved from the sequence read analysis. (**B**) Nucleotide sequences of the KKRR motif of these mole-rat species. (**C**) Sequence reads from the sequence read archive (SRA) database, which were retrieved by performing sequence read nucleotide BLAST with the exon three nucleotide sequence of the naked mole-rat used as the query sequence. Data are presented as % sequence reads that match the human KKRR nucleotide sequence 'aagaagagacgg'. Where possible, reads were retrieved from at least three individuals, from three separate studies. See Supplementary Data 2. Synonymous (green), non-synonymous (red), and non-synonymous to K/R (orange) mutations are highlighted. Model tree was manually generated using divergence times retrieved from TimeTree[3].

A

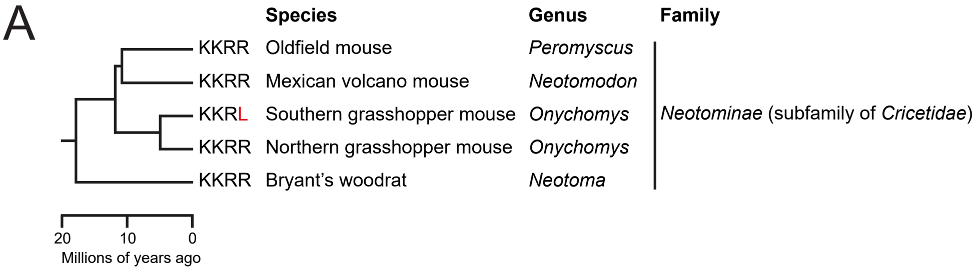

B

Species	Nucleotide sequence												Motif	NLS score
	K			K			R			R			KKRR	
Oldfield mouse	a	a	g	a	a	g	a	g	a	c	g	g	KKRR	10.5
Mexican volcano mouse	a	a	g	a	a	g	a	g	a	c	g	g	KKRR	10.5
Southern grasshopper mouse	a	a	g	a	a	g	a	g	a	c	t	g	KKRL	<2
Northern grasshopper mouse	a	a	g	a	a	g	a	g	a	c	g	g	KKRR	9
Bryant's woodrat	a	a	g	a	a	g	a	g	a	c	g	g	KKRR	9.5

match to human non-synonymous

C

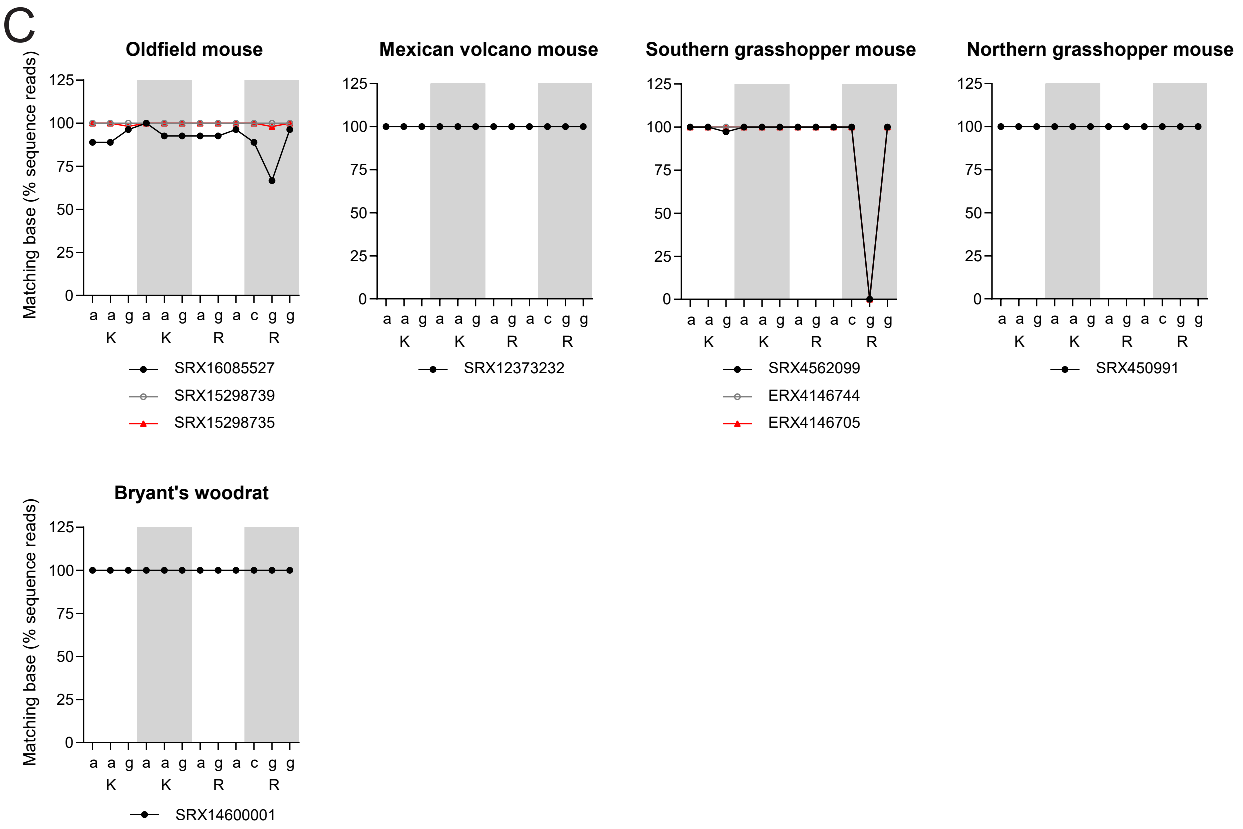

Supplementary Figure 9. Assessment of nuclear localisation sequence (NLS) mutations in the southern grasshopper mouse family. (**A**) Model evolutionary tree of the southern grasshopper mouse family, only including species for which an *IL1A* sequence could be retrieved from the sequence read analysis. (**B**) Nucleotide sequences of the KKRR motif of these species. (**C**) Sequence reads from the sequence read archive (SRA) database, which were retrieved by performing sequence read nucleotide BLAST with the exon three nucleotide sequence of the southern grasshopper mouse used as the query sequence. Data are presented as % sequence reads that match the human KKRR nucleotide sequence 'aagaagagacgg'. Where possible, reads were retrieved from at least three individuals, from three separate studies. See Supplementary Data 2. Synonymous (green) and non-synonymous (red) mutations are highlighted. Model tree was manually generated using divergence times retrieved from TimeTree[3].

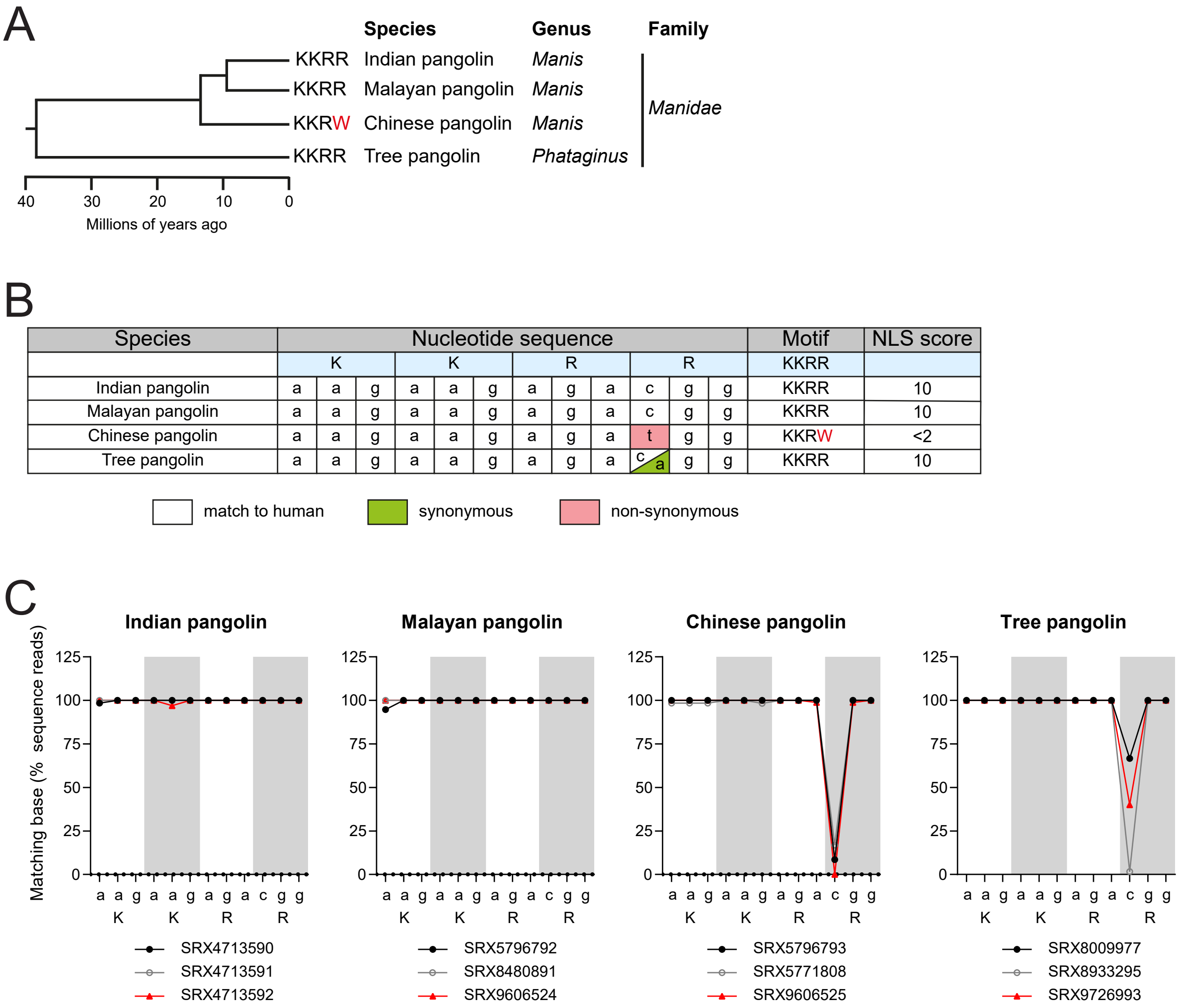

Supplementary Figure 10. Assessment of nuclear localisation sequence (NLS) mutations in the pangolin family. (**A**) Model evolutionary tree of the pangolin family. (**B**) Nucleotide sequences of the KKRR motif of these species for which an *IL1A* sequence could be retrieved from the sequence read analysis. (**C**) Sequence reads from the sequence read archive (SRA) database, which were retrieved by performing sequence read nucleotide BLAST with the exon three nucleotide sequence of the Chinese pangolin used as the query sequence. Data are presented as % sequence reads that match the human KKRR nucleotide sequence ‘aagaagagacgg’. Where possible, reads were retrieved from at least three individuals, from three separate studies. See Supplementary Data 2. Synonymous (green) and non-synonymous (red) mutations are highlighted. Model tree was manually generated using divergence times retrieved from TimeTree[3].

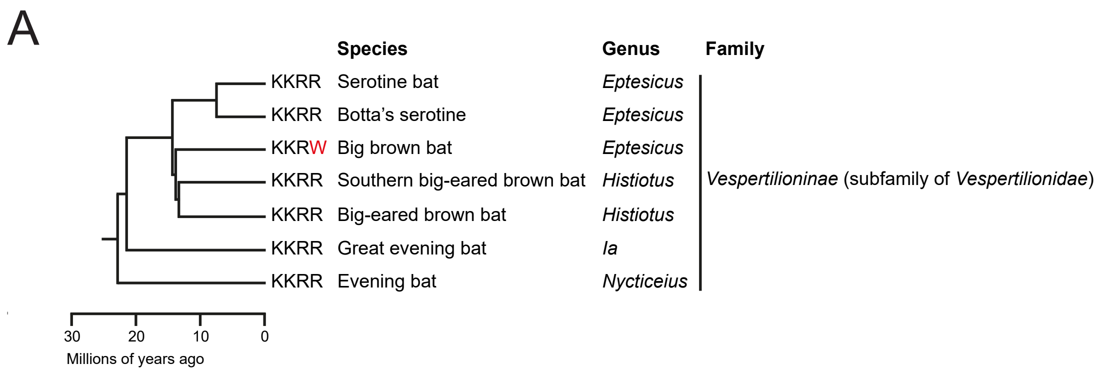

B

Species	Nucleotide sequence												Motif	NLS score
	K			K			R			R			KKRR	
Serotine bat	a	a	g	a	a	g	a	g	a	c	g	g / a	KKRR	10.5
Botta's serotine	a	a	g	a	a	g	a	g	a	c	g	g	KKRR	14.5
Big brown bat	a	a	g	a	a	g	a	g	a	c / t	g	g	KKRW	<2
Southern big-eared brown bat	a	a	g	a	a	g	a	g	a	c	g	g	KKRR	11
Big-eared brown bat	a	a	g	a	a	g	a	g	a	c	g	g	KKRR	11
Great evening bat	a	a	g	a	a	g	a	g	a	c	g	g	KKRR	10.5
Evening bat	a	a	g	a	a	g	a	g	a	c	g	g	KKRR	10.5

match to human | synonymous | non-synonymous

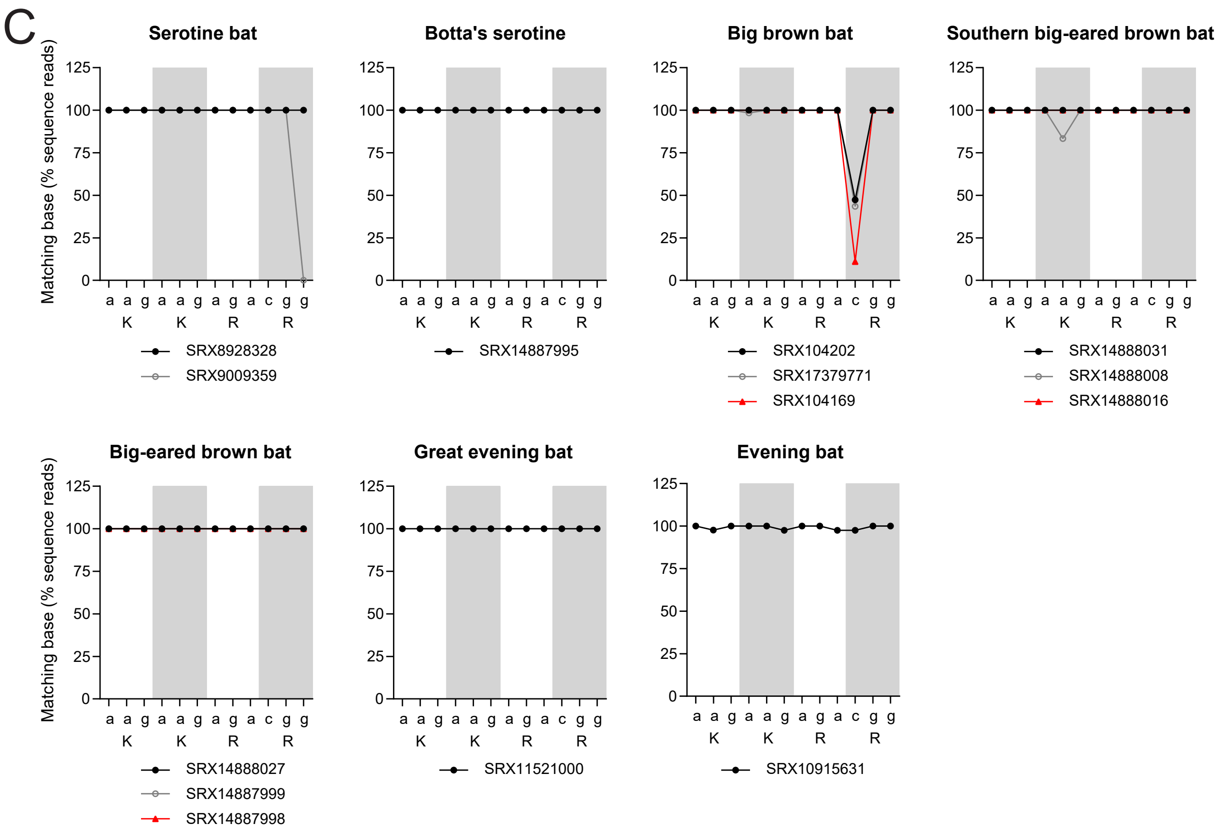

Supplementary Figure 11. Assessment of nuclear localisation sequence (NLS) mutations in the big brown bat family. (**A**) Model evolutionary tree of the big brown bat family, only including species for which an *IL1A* sequence could be retrieved from the sequence read analysis. (**B**) Nucleotide sequences of the KKRR motif of these species. (**C**) Sequence reads from the sequence read archive (SRA) database, which were retrieved by performing sequence read nucleotide BLAST with the exon three nucleotide sequence of the big brown bat used as the query sequence. Data are presented as % sequence reads that match the human KKRR nucleotide sequence 'aagaagagacgg'. Where possible, reads were retrieved from at least three individuals, from three separate studies. See Supplementary Data 2. Synonymous (green) and non-synonymous (red) mutations are highlighted. Model tree was manually generated using divergence times retrieved from TimeTree[3].

Species	Nucleotide sequence												Motif	NLS score
	K			K			R			R			KKRR	
Grey short-tailed opossum	a	a	g	a	a	g	a	g	g	c	g	t	KKRR	8
Virginia opossum	a	a	g	a	a	g	a	g	g	t	g	t	KKRC	6.5
Monito del Monte	a	a	a	g	a	g	a	g	a	a	g	a	KERR	<2
Koala	a	a	g	a	a	g	a	g	a	c	a	a	KKRQ	<2
Common wombat	a	a	g	a	a	g	a	g	a	c	a	a	KKRQ	<2
Coppery ringtail possum	a	a	g	g	a	g	a	g	a	a	g	a	KERR	<2
Western ringtail possum	a	a	g	g	a	g	a	g	a	a	g	a	KERR	<2
Swamp wallaby	a	a	g	a	a	g	a	g	a	c	a	a	KKRQ	<2
Western brush wallaby	a	a	g	a	a	g	a	g	a	c	a	a	KKRQ	<2
Red kangaroo	a	a	g	a	a	g	a	g	a	c	a	a	KKRQ	<2
Western grey kangaroo	a	a	g	a	a	g	a	g	a	c	a	a	KKRQ	<2
Rufous hare-wallaby	g	t	a	a	a	g	a	g	a	c	a	a	VKRQ	<2
Quokka	a	a	g	a	a	g	a	g	a	c	a	a	KKRQ	<2
Yellow-footed rock-wallaby	a	a	g	a	a	g	a	g	a	c	a	a	KKRQ	<2
Matschie’s tree-kangaroo	a	a	g	a	a	g	a	g	a	c	a	a	KKRQ	<2
Woylie	a	a	g	a	a	a	a	g	a	c	a	a	KKRQ	<2
Gilbert’s potoroo	a	a	g	a	a	g	a	g	a	c	a	a	KKRQ	<2
Common brushtail	g	a	a	a	a	t	a	g	a	c	g	a	ENRR	<2
Ground cuscus	g	a	a	a	a	t	a	g	a	c	g	a	ENRR	<2
Southern marsupial mole	a	a	g	a	a	g	a	g	c	c	a	a	KKSQ	<2
Eastern quoll	a	a	g	a	a	g	a	a	a	c	a	a	KKKQ	<2
Tasmanian devil	a	a	g	a	a	g	a	a	a	c	a	a	KKKQ	3
Brush-tailed phascogale	a	a	g	a	a	g	a	a	a	c	a	a	KKKQ	<2
Brown antechinus	a	a	g	a	a	g	a	a	a	g	a	a	KKKE	<2
Fat-tailed dunnart	a	a	g	a	a	g	a	a	a	c	a	a	KKKQ	<2
Tasmanian tiger	a	a	g	a	a	g	a	g	a	c	g	a	KKRR	12

- match to human
- synonymous
- non-synonymous to K/R
- non-synonymous

Supplementary Figure 12. Assessment of nuclear localisation sequence (NLS) mutations in the marsupial lineage. Nucleotide sequences of the KKRR motif of these species. Synonymous (green), non-synonymous (red), and non-synonymous to K/R (orange) mutations are highlighted.

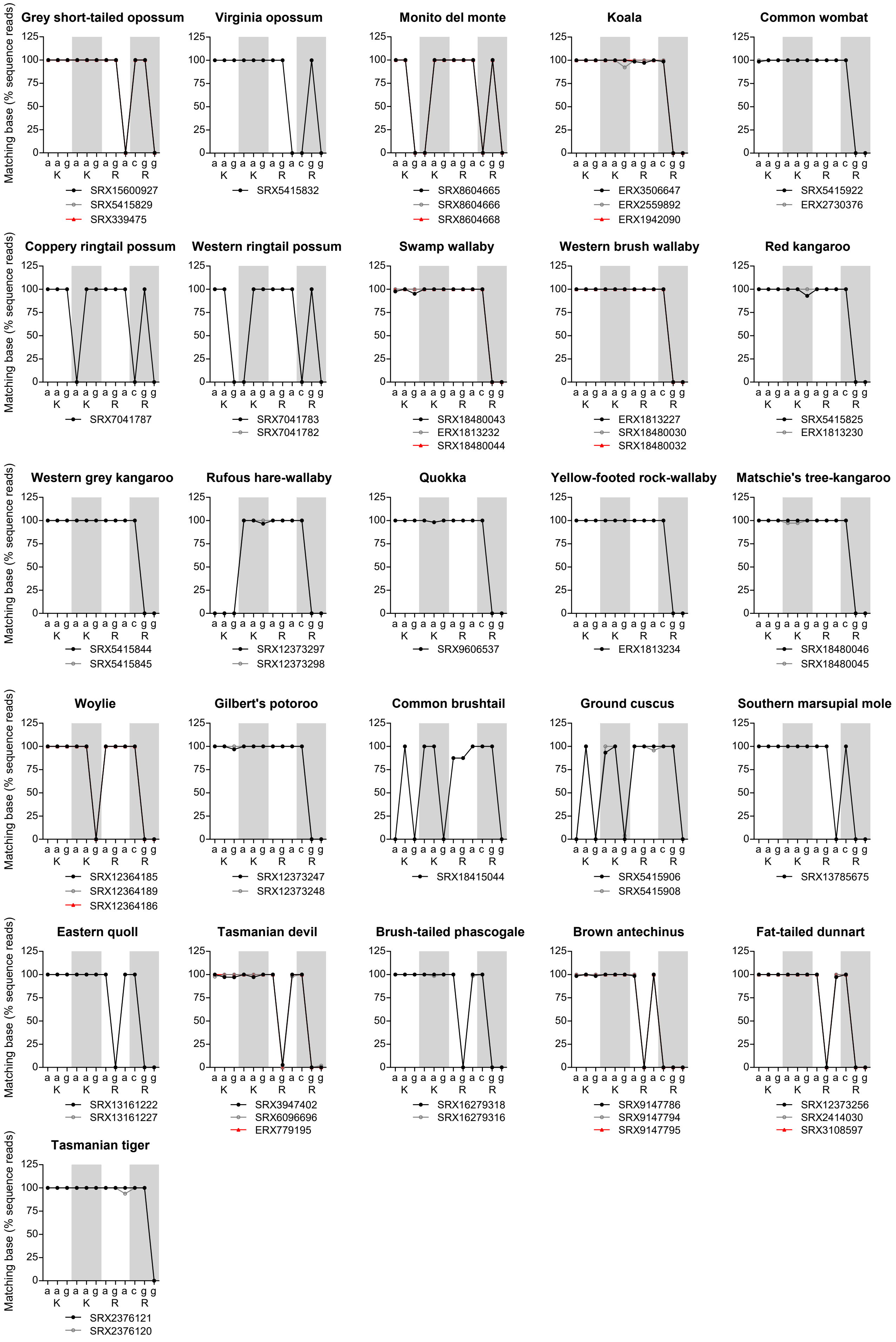

Matching base (% sequence reads)
125
100
75
50
25
0
a a g a a g a g a c g g
K K R R
Grey short-tailed opossum
SRX15600927
SRX5415829
SRX339475
Virginia opossum
SRX5415832
Monito del monte
SRX8604665
SRX8604666
SRX8604668
Koala
ERX3506647
ERX2559892
ERX1942090
Common wombat
SRX5415922
ERX2730376
Coppery ringtail possum
SRX7041787
Western ringtail possum
SRX7041783
SRX7041782
Swamp wallaby
SRX18480043
ERX1813232
SRX18480044
Western brush wallaby
ERX1813227
SRX18480030
SRX18480032
Red kangaroo
SRX5415825
ERX1813230
Western grey kangaroo
SRX5415844
SRX5415845
Rufous hare-wallaby
SRX12373297
SRX12373298
Quokka
SRX9606537
Yellow-footed rock-wallaby
ERX1813234
Matschie's tree-kangaroo
SRX18480046
SRX18480045
Woylie
SRX12364185
SRX12364189
SRX12364186
Gilbert's potoroo
SRX12373247
SRX12373248
Common brushtail
SRX18415044
Ground cuscus
SRX5415906
SRX5415908
Southern marsupial mole
SRX13785675
Eastern quoll
SRX13161222
SRX13161227
Tasmanian devil
SRX3947402
SRX6096696
ERX779195
Brush-tailed phascogale
SRX16279318
SRX16279316
Brown antechinus
SRX9147786
SRX9147794
SRX9147795
Fat-tailed dunnart
SRX12373256
SRX2414030
SRX3108597
Tasmanian tiger
SRX2376121
SRX2376120

Supplementary Figure 13. Validation of nuclear localisation sequence (NLS) mutations in the marsupial lineage. Sequence reads from the sequence read archive (SRA) database, which were retrieved by performing sequence read nucleotide BLAST with the exon three nucleotide sequence of the koala, Tasmanian devil or common brushtail used as the query sequence. Data are presented as % sequence reads that match the human KKRR nucleotide sequence 'aagaagagacgg'. Where possible, reads were retrieved from at least three individuals, from three separate studies. See Supplementary Data 2.

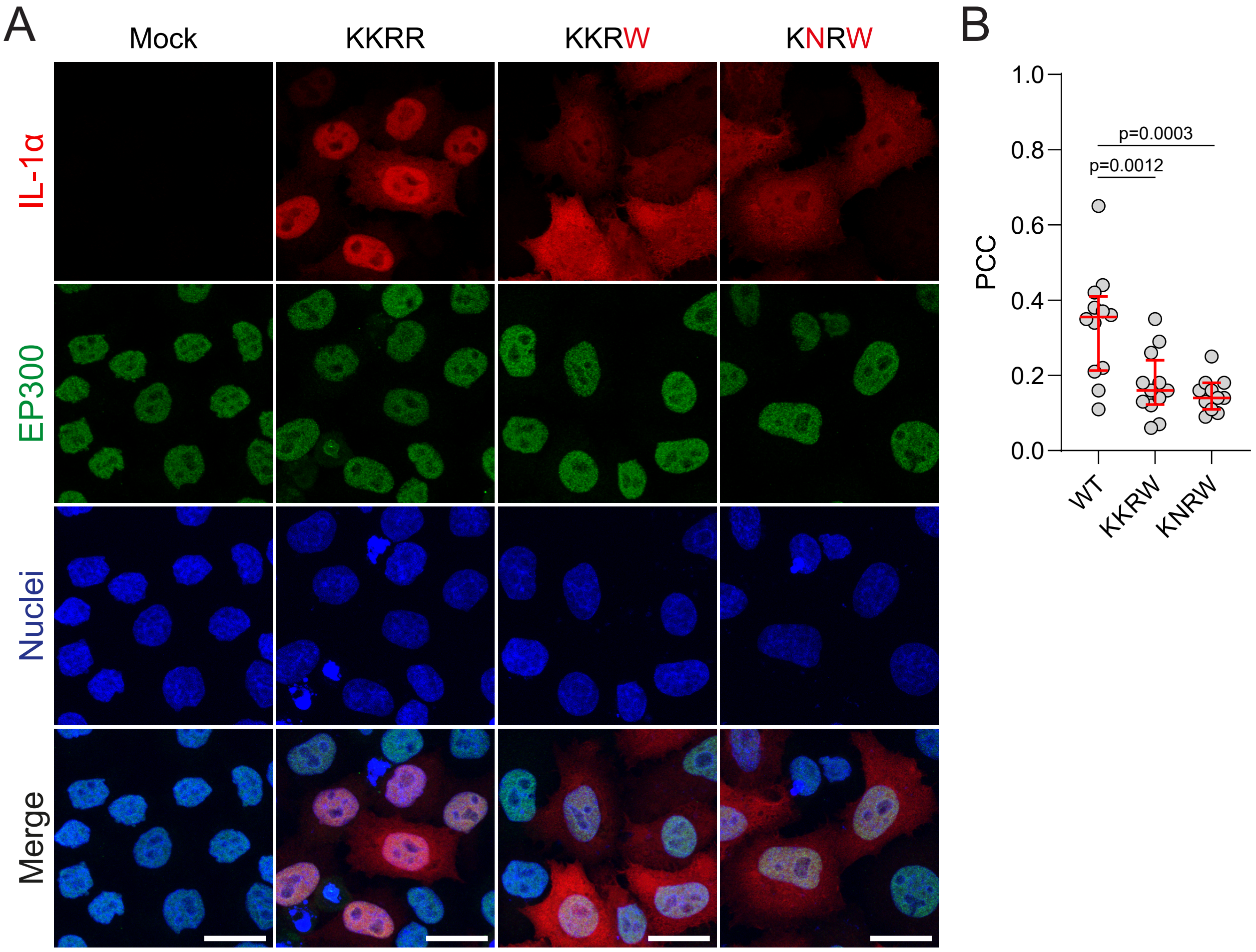

Supplementary Figure 14. Loss of pro-IL-1α nuclear localisation reduces co-localisation with EP300. HeLa cells were transfected with human pro-IL-1α plasmids containing a KKRR, KKRW, or KNRW nuclear localisation sequence (NLS) motif. (**A**) Representative maximum projection confocal immunofluorescence images are shown (n=11-12 fields of view from four independent experiments). (**B**) Co-localisation of IL-1α signal with EP300 was determined by Pearson's correlation coefficient (PCC). Scale bars are 20 μm. Data are median ± IQR. Data were analysed using one-way ANOVA followed by Dunnett's post-hoc test (B). Source data are provided as a Source Data file.

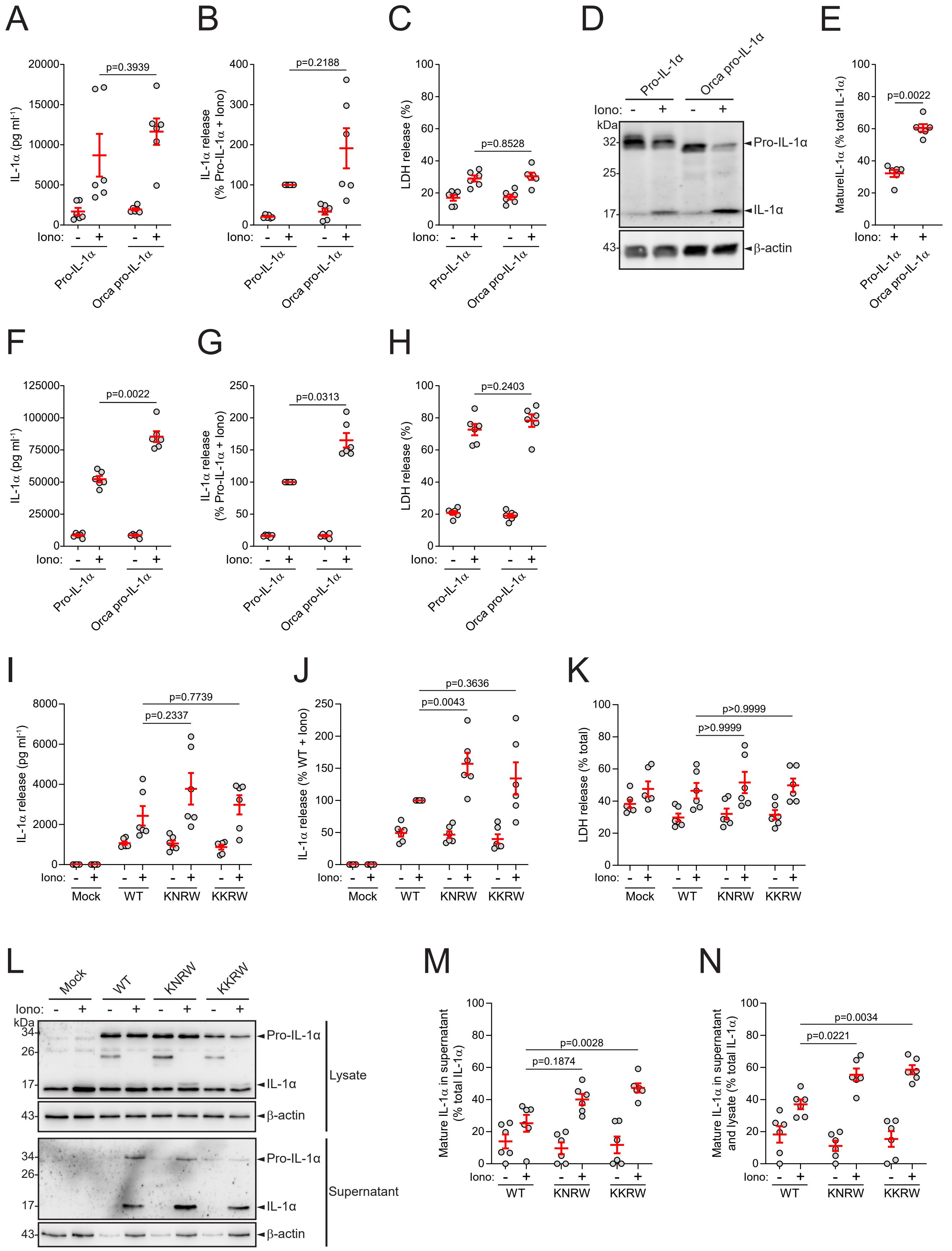
A
B
C
D
E
F
G
H
I
J
K
L
M
N
IL-1α (pg ml-1)
IL-1α release (% Pro-IL-1α + Iono)
LDH release (%)
MatureIL-1α (% total IL-1α)
IL-1α release (pg ml-1)
IL-1α release (% WT + Iono)
LDH release (% total)
Mature IL-1α in supernatant (% total IL-1α)
Mature IL-1α in supernatant and lysate (% total IL-1α)
Iono:
Pro-IL-1α
Orca pro-IL-1α
Mock
WT
KNRW
KKRW
p=0.3939
p=0.2188
p=0.8528
p=0.0022
p=0.0022
p=0.0313
p=0.2403
p=0.7739
p=0.2337
p=0.3636
p=0.0043
p>0.9999
p>0.9999
p=0.0028
p=0.1874
p=0.0034
p=0.0221
kDa
IL-1α
β-actin
Lysate
Supernatant

Supplementary Figure 15. Reduction of pro-IL-1α nuclear localisation enhances IL-1α cleavage and release. (**A-H**) HeLa cells were transfected with full length human pro-IL-1α or Orca IL-1α pro-domain fused to the human IL-1α mature domain (Orca pro-IL-1α) for 18 h and then were treated with ionomycin (10 μM) for 1 h (A-E) or 4 h (F-H) (n=6). (A, F) IL-1α release was measured by ELISA, and (B, G) expressed relative to human pro-IL-1α that received ionomycin treatment. (C, H) Cell death was determined by measuring lactate dehydrogenase (LDH) release into the supernatant. (D) Combined supernatants and lysates were analysed for pro-IL-1α cleavage by western blotting, and (E) densitometry was performed to determine the ratio of mature IL-1α to total IL-1α. (**I-N**) HeLa cells were transfected with human pro-IL-1α plasmids containing a WT (KKRR), KNRW, or KKRW NLS motif for 24 h, and treated with ionomycin (10 μM) for 1 h (n=6). (I) IL-1α release was measured by ELISA, and (J) expressed relative to WT pro-IL-1α that received ionomycin treatment. (K) Cell death was determined by measuring LDH release into the supernatant. (L) Supernatants and lysates were individually analysed for pro-IL-1α cleavage by western blotting and densitometry was performed to determine the ratio of (M) mature IL-1α in the supernatant to total IL-1α or (N) mature IL-1α in the supernatant and lysate to total IL-1α. Data are mean ± SEM. Data were analysed using unpaired two-tailed Mann-Whitney test (pro-IL-1α + ionomycin versus orca pro-IL-1α + ionomycin) (A, C, E, F, H), Wilcoxon signed-rank test versus a hypothetical value of 100% (pro-IL-1α + ionomycin versus orca pro-IL-1α + ionomycin) (B, G), Kruskal-Wallis test followed by Dunn's post-hoc test (versus WT + ionomycin) (I, K, M, N), or multiple unpaired Mann-Whitney tests versus a value of 100% followed by Holm-Sidak correction (WT + ionomycin versus KNRW + ionomycin or KKRW + ionomycin) (J). Source data are provided as a Source Data file.

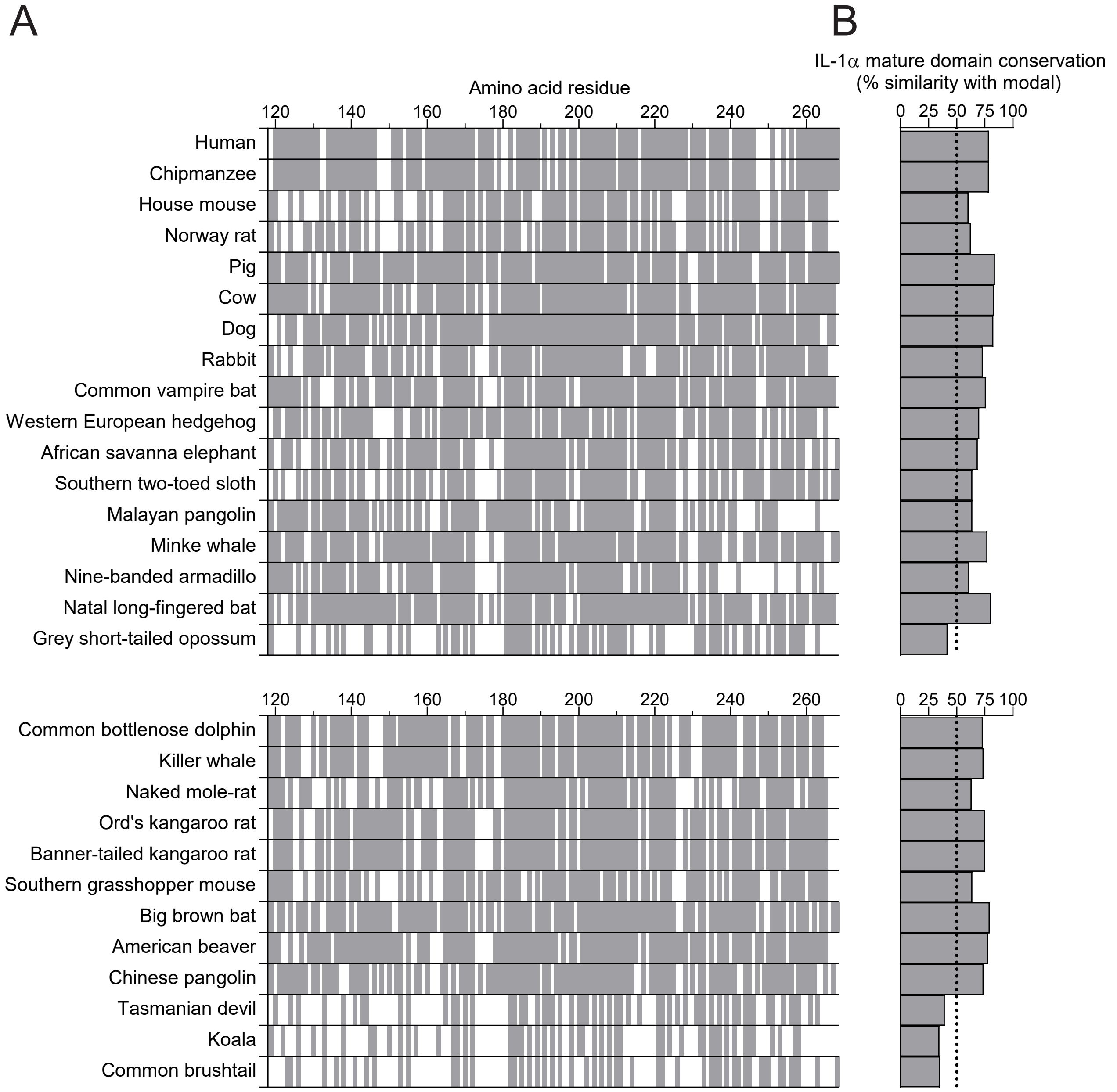

Supplementary Figure 16. Conservation of the IL-1α mature domain. (**A**) IL-1α mature domain amino acid conservation in representative intact nuclear localisation sequence (NLS) (top) and mutated NLS (bottom) species. Residues that match the modal amino acid from the full sequence alignment are indicated by a solid grey colour, whereas residues that did not match are indicated by a white gap. Positions where gaps were the modal residue were removed from the alignment. See Supplementary Data 1. (**B**) Conservation of the whole IL-1α mature domain relative to the modal mature domain sequence. Source data are provided as a Source Data file.

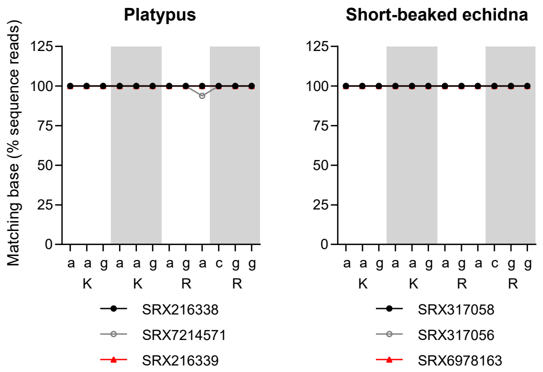

Supplementary Figure 17. Validation of NLS mutations in the monotreme lineage. Sequence reads from the sequence read archive (SRA) database, which were retrieved by performing sequence read nucleotide BLAST with the exon three nucleotide sequence of the platypus or short-beaked echidna used as the query sequence. Data are presented as % sequence reads that match the human KKRR nucleotide sequence 'aagaagagacgg'. Where possible, reads were retrieved from at least three individuals, from three separate studies. See Supplementary Data 2.

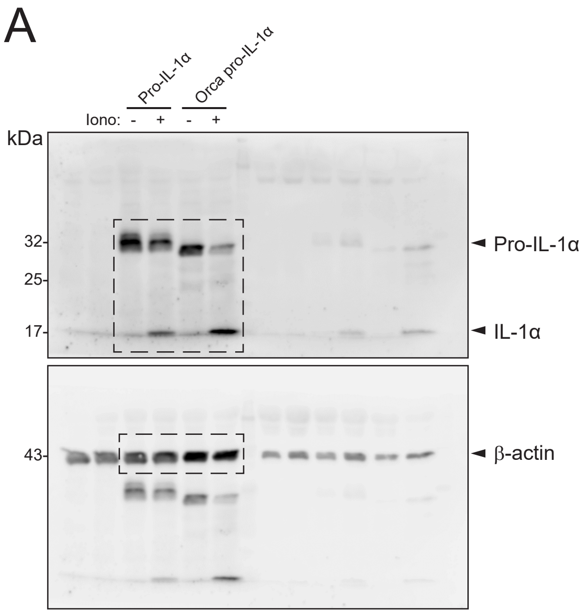

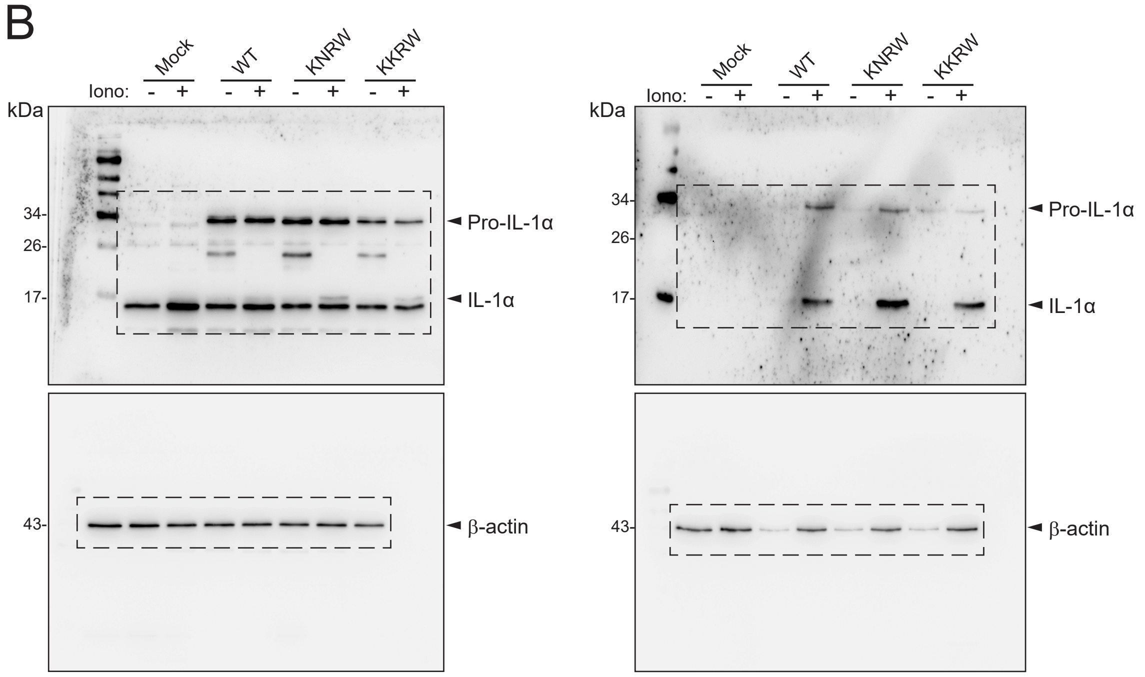

Supplementary Figure 18. Uncropped blots from Supplementary Figure 15. Uncropped blots from (**A**) Supplementary Figure 15D and (**B**) Supplementary Figure 15L.

Supplementary References

1 Jones, D. T., Taylor, W. R. & Thornton, J. M. The rapid generation of mutation data matrices from protein sequences. *Comput Appl Biosci* **8**, 275-282 (1992). https://doi.org:10.1093/bioinformatics/8.3.275

2 Felsenstein, J. CONFIDENCE LIMITS ON PHYLOGENIES: AN APPROACH USING THE BOOTSTRAP. *Evolution* **39**, 783-791 (1985). https://doi.org:10.1111/j.1558-5646.1985.tb00420.x

3 Kumar, S. *et al*. TimeTree 5: An Expanded Resource for Species Divergence Times. *Molecular biology and evolution* **39** (2022). https://doi.org:10.1093/molbev/msac174
