# Supplementary material for: Proximity labelling of pro-interleukin-1α reveals evolutionary conserved nuclear interactions
Source: Nat Commun. 2024 Aug 8;15:6750. doi: 10.1038/s41467-024-50901-0 (PMC11310415; doi:10.1038/s41467-024-50901-0)
Supplement: Supplementary file 3 — Description of Additional Supplementary Files [file 41467_2024_50901_MOESM3_ESM.pdf]

Description of Additional Supplementary Files

File Name: Supplementary Data 1
Description: IL-1α amino acid sequence alignment in mammals and monotremes, including sequence accession details.

File Name: Supplementary Data 2
Description: Sequence read archive (SRA) accession details and alignments of KKRR motif nucleotide sequence.

File Name: Supplementary Data 3
Description: Full plasmid sequences and primer sequences.
