# Supplementary material for: Proximity labelling of pro-interleukin-1α reveals evolutionary conserved nuclear interactions
Source: Nat Commun. 2024 Aug 8;15:6750. doi: 10.1038/s41467-024-50901-0 (PMC11310415; doi:10.1038/s41467-024-50901-0)
Supplement: Supplementary file 7 — Reporting Summary [file 41467_2024_50901_MOESM7_ESM.pdf]

nature portfolio

Corresponding author(s): Christopher Hoyle, David Brough

Last updated by author(s): Apr 4, 2024

Reporting Summary

Nature Portfolio wishes to improve the reproducibility of the work that we publish. This form provides structure for consistency and transparency in reporting. For further information on Nature Portfolio policies, see our Editorial Policies and the Editorial Policy Checklist.

Statistics

For all statistical analyses, confirm that the following items are present in the figure legend, table legend, main text, or Methods section.

n/a	Confirmed	
☐	☒	The exact sample size (*n*) for each experimental group/condition, given as a discrete number and unit of measurement
☒	☐	A statement on whether measurements were taken from distinct samples or whether the same sample was measured repeatedly
☐	☒	The statistical test(s) used AND whether they are one- or two-sided<br>*Only common tests should be described solely by name; describe more complex techniques in the Methods section.*
☒	☐	A description of all covariates tested
☐	☒	A description of any assumptions or corrections, such as tests of normality and adjustment for multiple comparisons
☐	☒	A full description of the statistical parameters including central tendency (e.g. means) or other basic estimates (e.g. regression coefficient) AND variation (e.g. standard deviation) or associated estimates of uncertainty (e.g. confidence intervals)
☐	☒	For null hypothesis testing, the test statistic (e.g. *F*, *t*, *r*) with confidence intervals, effect sizes, degrees of freedom and *P* value noted<br>*Give P values as exact values whenever suitable.*
☒	☐	For Bayesian analysis, information on the choice of priors and Markov chain Monte Carlo settings
☒	☐	For hierarchical and complex designs, identification of the appropriate level for tests and full reporting of outcomes
☐	☒	Estimates of effect sizes (e.g. Cohen's *d*, Pearson's *r*), indicating how they were calculated

Our web collection on statistics for biologists contains articles on many of the points above.

Software and code

Policy information about availability of computer code

Data collection	NCBI orthologue database (https://www.ncbi.nlm.nih.gov/gene/3552/ortholog/)<br>Sequence read archive (https://www.ncbi.nlm.nih.gov/sra)<br>Micromanager software v1.4.23<br>LAS X software (v3.5.1.18803)<br>Genesys software<br>Sequence read archive nucleotide BLAST (https://blast.ncbi.nlm.nih.gov/Blast.cgi?PAGE=MegaBlast&PROGRAM=blastn&BLAST_PROGRAMS=megaBlast&PAGE_TYPE=BlastSearch&BLAST_SPEC=SRA)
Data analysis	Perseus computational platform (Tyanova et al., 2016; https://maxquant.net/perseus/)<br>Ingenuity pathway analysis (IPA, Qiagen)<br>STRING (https://string-db.org/)<br>MegaX<br>NLSmapper (https://nls-mapper.iab.keio.ac.jp/)<br>ImageJ<br>MaxQuant software (v1.6.11.0)<br>Expasy translate (https://web.expasy.org/translate/)<br>GraphPad Prism (v9)

For manuscripts utilizing custom algorithms or software that are central to the research but not yet described in published literature, software must be made available to editors and reviewers. We strongly encourage code deposition in a community repository (e.g. GitHub). See the Nature Portfolio guidelines for submitting code & software for further information.

Data

Policy information about availability of data

All manuscripts must include a data availability statement. This statement should provide the following information, where applicable:

- Accession codes, unique identifiers, or web links for publicly available datasets
- A description of any restrictions on data availability
- For clinical datasets or third party data, please ensure that the statement adheres to our policy

The data that support the findings of this study are available within the article, supplementary figures and supplementary data files. Source data are provided with this paper. Uncropped western blots are provided in the source data file and in Supplementary Figure 18. Proteomics mass spectrometry raw data are available at the ProteomeXchange Consortium via the PRIDE partner repository under the accession code PXD053438 (https://www.ebi.ac.uk/pride/archive/projects/PXD053438), and processed log2-transformed LFQ values are available in the source data file. All IL-1α amino acid sequence accession codes are listed in Supplementary Data 1. All sequence read archive accession codes are listed in Supplementary Data 2.

Research involving human participants, their data, or biological material

Policy information about studies with human participants or human data. See also policy information about sex, gender (identity/presentation), and sexual orientation and race, ethnicity and racism.

Reporting on sex and gender	*Use the terms sex (biological attribute) and gender (shaped by social and cultural circumstances) carefully in order to avoid confusing both terms. Indicate if findings apply to only one sex or gender; describe whether sex and gender were considered in study design; whether sex and/or gender was determined based on self-reporting or assigned and methods used.* *Provide in the source data disaggregated sex and gender data, where this information has been collected, and if consent has been obtained for sharing of individual-level data; provide overall numbers in this Reporting Summary. Please state if this information has not been collected.* *Report sex- and gender-based analyses where performed, justify reasons for lack of sex- and gender-based analysis.*
Reporting on race, ethnicity, or other socially relevant groupings	*Please specify the socially constructed or socially relevant categorization variable(s) used in your manuscript and explain why they were used. Please note that such variables should not be used as proxies for other socially constructed/relevant variables (for example, race or ethnicity should not be used as a proxy for socioeconomic status).* *Provide clear definitions of the relevant terms used, how they were provided (by the participants/respondents, the researchers, or third parties), and the method(s) used to classify people into the different categories (e.g. self-report, census or administrative data, social media data, etc.)* *Please provide details about how you controlled for confounding variables in your analyses.*
Population characteristics	*Describe the covariate-relevant population characteristics of the human research participants (e.g. age, genotypic information, past and current diagnosis and treatment categories). If you filled out the behavioural & social sciences study design questions and have nothing to add here, write "See above."*
Recruitment	*Describe how participants were recruited. Outline any potential self-selection bias or other biases that may be present and how these are likely to impact results.*
Ethics oversight	*Identify the organization(s) that approved the study protocol.*

Note that full information on the approval of the study protocol must also be provided in the manuscript.

Field-specific reporting

Please select the one below that is the best fit for your research. If you are not sure, read the appropriate sections before making your selection.

[x] Life sciences   [ ] Behavioural & social sciences   [ ] Ecological, evolutionary & environmental sciences

For a reference copy of the document with all sections, see nature.com/documents/nr-reporting-summary-flat.pdf

Life sciences study design

All studies must disclose on these points even when the disclosure is negative.

Sample size	Three to six independent biological replicates were performed for each experiment. Multiple fields of view were used for each independent biological replicate during immunofluorescence image analysis. A minimum sample size of three was chosen with no sample size calculations performed.
Data exclusions	No data were excluded.
Replication	Three to six independent biological replicates were performed for each experiment. All attempts at replication were successful, and all individual data points are shown in graphs. All data was collected from matched samples to account for covariates between independent replicates

Randomization	None.
Blinding	Experimenters were blinded during immunofluorescence image acquisition and analysis when appropriate. No blinding was performed for all other experiments.

Reporting for specific materials, systems and methods

We require information from authors about some types of materials, experimental systems and methods used in many studies. Here, indicate whether each material, system or method listed is relevant to your study. If you are not sure if a list item applies to your research, read the appropriate section before selecting a response.

Materials & experimental systems

n/a	Involved in the study	
☐	☒	Antibodies
☐	☒	Eukaryotic cell lines
☒	☐	Palaeontology and archaeology
☐	☒	Animals and other organisms
☒	☐	Clinical data
☒	☐	Dual use research of concern
☒	☐	Plants

Methods

n/a	Involved in the study	
☒	☐	ChIP-seq
☒	☐	Flow cytometry
☒	☐	MRI-based neuroimaging

Antibodies

Antibodies used

Primary Antibodies

Goat anti-human IL-1α (AF-200-NA) (lot: AAB0522021) (IF: 2 µg ml-1, WB: 200 ng ml-1) (R&D)
Goat anti-mouse IL-1α (AF-400-NA) (lot: AMG0219011) (IF: 2 µg ml-1) (R&D)
Rabbit anti-human BirA (11582-RP01) (lot: HB070C1213-B) (IF: 1:500 v/v, WB: 1:1000 v/v) (Sino Biological)
Anti-β-actin-peroxidase (A3854) (lot: 0000239225) (WB: 1:20000 v/v) (Sigma-Aldrich)
Rabbit anti-human ZZZ3 (PA5-90723) (lot: YK4107715B) (IF: 9.3 µg ml-1, WB: 930 ng ml-1) (Invitrogen)
Streptavidin-HRP conjugate (S911) (lot: 2384052) (IF: 1 µg ml-1, WB: 500 ng ml-1) (Invitrogen)
Streptavidin Alexa Fluor™ 594 conjugate (S11227) (IF: 4 µg ml-1) (Invitrogen)
Rabbit anti-human NCAPH (11515-1-AP) (lot: 00043709) (WB: 600 ng ml-1) (Proteintech)
Rabbit anti-human EP300 (ab275378) (lot: 1009869-21) (IF: 4.6 µg ml-1, WB: 460 ng ml-1) (Abcam)
Rabbit anti-human ANKRD17 (HPA063731) (lot: R88801) (IF: 2 µg ml-1) (Atlas antibodies)

Secondary Antibodies

Alexa Fluor™ 488 donkey anti-goat IgG (A-11055) (lot: 2604365) (IF: 4 µg ml-1) (Invitrogen)
Alexa Fluor™ 594 donkey anti-goat IgG (A-11058) (lot: 2641989) (IF: 4 µg ml-1) (Invitrogen)
Alexa Fluor 647™ donkey anti-rabbit IgG (A-31573) (lot: TH271042) (IF: 4 µg ml-1) (Invitrogen)
Rabbit anti-goat IgG (P044901-2) (lot: 41628067) (WB: 500 ng ml-1) (Agilent)
Goat anti-rabbit IgG (P044801-2) (lot: 41653919) (WB: 1000 ng ml-1) (Agilent)

Validation

Secondary antibody only controls, or untreated or untransfected cells were used for immunofluorescence labelling validation. Untreated or untransfected cells validated primary antibodies used for western blotting, combined with molecular weight.

Validation statements available from manufacturers:
Goat anti-human IL-1α (AF-200-NA) https://www.rndsystems.com/products/human-il-1alpha-il-1f1-antibody_af-200-na
Goat anti-mouse IL-1α (AF-400-NA) https://www.rndsystems.com/products/mouse-il-1alpha-il-1f1-antibody_af-400-na
Rabbit anti-human BirA (11582-RP01) https://www.sinobiological.com/antibodies/bira-11582-rp01
Anti-β-actin-peroxidase (A3854) https://www.sigmaaldrich.com/GB/en/product/sigma/a3854?utm_source=google&utm_medium=cpc&utm_campaign=15001183131&utm_content=127306766063&gclid=Cj0KCQjw4MSzBhC8ARIsAPFOuyXOlax-vjUqZ00b9vp8dW7aZI2-Ck28Dk1dhXb0NV0jt_guwAvRYnIaApPFEALw_wcB
Rabbit anti-human ZZZ3 (PA5-90723) https://www.thermofisher.com/antibody/product/ZZZ3-Antibody-Polyclonal/PA5-90723
Streptavidin-HRP conjugate (S911) https://www.thermofisher.com/order/catalog/product/S911
Streptavidin Alexa Fluor™ 594 conjugate (S11227) https://www.thermofisher.com/order/catalog/product/S11227?SID=srch-srp-S11227
Rabbit anti-human NCAPH (11515-1-AP) https://www.ptglab.com/products/NCAPH-Antibody-11515-1-AP.htm
Rabbit anti-human EP300 (ab275378) https://www.abcam.com/en-gb/products/primary-antibodies/kat3b-p300-antibody-epr23495-268-chip-grade-ab275378
Rabbit anti-human ANKRD17 (HPA063731) https://www.atlasantibodies.com/products/primary-antibodies/triple-a-polyclonals/anti-ankrd17-antibody-hpa063731/
Alexa Fluor™ 488 donkey anti-goat IgG (A-11055) https://www.thermofisher.com/antibody/product/Donkey-anti-Goat-IgG-H-L-Cross-Adsorbed-Secondary-Antibody-Polyclonal/A-11055

Alexa Fluor™ 594 donkey anti-goat IgG (A-11058) https://www.thermofisher.com/antibody/product/Donkey-anti-Goat-IgG-H-L-Cross-Adsorbed-Secondary-Antibody-Polyclonal/A-11058
Alexa Fluor 647™ donkey anti-rabbit IgG (A-31573) https://www.thermofisher.com/antibody/product/Donkey-anti-Rabbit-IgG-H-L-Highly-Cross-Adsorbed-Secondary-Antibody-Polyclonal/A-31573
Rabbit anti-goat IgG (P044901-2) https://www.agilent.com/store/en_US/Prod-P044901-2/P044901-2
Goat anti-rabbit IgG (P044801-2) https://www.agilent.com/store/en_US/Prod-P044801-2/P044801-2

Eukaryotic cell lines

Policy information about cell lines and Sex and Gender in Research

Cell line source(s)	HeLa (ATCC), THP-1 (ATCC)
Authentication	Neither of the cell lines were recently authenticated
Mycoplasma contamination	Cell lines were routinely tested for mycoplasma contamination
Commonly misidentified lines (See ICLAC register)	None

Animals and other research organisms

Policy information about studies involving animals; ARRIVE guidelines recommended for reporting animal research, and Sex and Gender in Research

Laboratory animals	Wild-type C57BL6/J mice, 3-6 months old, Charles River
Wild animals	N/A
Reporting on sex	Male and female mice used for bone marrow collection
Field-collected samples	N/A
Ethics oversight	All procedures adhered to the UK Animals (Scientific Procedures) Act (1986) and were approved by the local Animal Ethical Review Group, The University of Manchester, with appropriate personal and project licences in place.

Note that full information on the approval of the study protocol must also be provided in the manuscript.

Plants

Seed stocks	*Report on the source of all seed stocks or other plant material used. If applicable, state the seed stock centre and catalogue number. If plant specimens were collected from the field, describe the collection location, date and sampling procedures.*
Novel plant genotypes	*Describe the methods by which all novel plant genotypes were produced. This includes those generated by transgenic approaches, gene editing, chemical/radiation-based mutagenesis and hybridization. For transgenic lines, describe the transformation method, the number of independent lines analyzed and the generation upon which experiments were performed. For gene-edited lines, describe the editor used, the endogenous sequence targeted for editing, the targeting guide RNA sequence (if applicable) and how the editor was applied.*
Authentication	*Describe any authentication procedures for each seed stock used or novel genotype generated. Describe any experiments used to assess the effect of a mutation and, where applicable, how potential secondary effects (e.g. second site T-DNA insertions, mosiacism, off-target gene editing) were examined.*
